# Transmembrane Fluoride Transport by a Cyclic Azapeptide With Two β-Turns

**DOI:** 10.3389/fchem.2020.621323

**Published:** 2021-01-12

**Authors:** Zhixing Zhao, Miaomiao Zhang, Bailing Tang, Peimin Weng, Yueyang Zhang, Xiaosheng Yan, Zhao Li, Yun-Bao Jiang

**Affiliations:** The MOE Key Laboratory of Spectrochemical Analysis and Instrumentation, Department of Chemistry, College of Chemistry and Chemical Engineering, Collaborative Innovation Center of Chemistry for Energy Materials (iChEM), Xiamen University, Xiamen, China

**Keywords:** cyclic azapeptide, β-turn, fluoride, ion recognition, transmembrane transport

## Abstract

Diverse classes of anion transporters have been developed, most of which focus on the transmembrane chloride transport due to its significance in living systems. Fluoride transport has, to some extent, been overlooked despite the importance of fluoride channels in bacterial survival. Here, we report the design and synthesis of a cyclic azapeptide (a peptide-based *N*-amidothiourea, **1**), as a transporter for fluoride transportation through a confined cavity that encapsulates fluoride, together with acyclic control compounds, the analogs **2** and **3**. Cyclic receptor **1** exhibits more stable β-turn structures than the control compounds **2** and **3** and affords a confined cavity containing multiple inner –NH protons that serve as hydrogen bond donors to bind anions. It is noteworthy that the cyclic receptor **1** shows the capacity to selectively transport fluoride across a lipid bilayer on the basis of the osmotic and fluoride ion-selective electrode (ISE) assays, during which an electrogenic anion transport mechanism is found operative, whereas no transmembrane transport activity was found with **2** and **3**, despite the fact that **2** and **3** are also able to bind fluoride via the thiourea moieties. These results demonstrate that the encapsulation of an anionic guest within a cyclic host compound is key to enhancing the anion transport activity and selectivity.

## Introduction

In the past decade, artificial transmembrane anion transport has attracted much attention, and a variety of synthetic carriers (Wu et al., 2018, 2019) or channels (Ren et al., 2018a,b) have been developed to facilitate the transport of anions, e.g., chloride (Valkenier et al., 2019), bicarbonate (Gale et al., 2010), and sulfate (Busschaert et al., 2014), across the cell membranes or artificial lipid bilayers, showcasing potential application in treating diseases caused by faulty anion channels, such as cystic fibrosis (also known as channelopathies; Sheppard et al., 1993), Bartter syndrome (Simon et al., 1996), and Dent's disease (Dutzler et al., 2002).

Fluoride is the smallest halide anion, which is closely related to the life and health of humans and other living organisms. For example, fluoride is an indispensable additive in toothpastes to prevent dental caries of children and adolescents (Marinho et al., 2003). However, long-term exposure to environments containing high-level fluoride will cause fluorosis (Zhang et al., 2017). Therefore, the concentration of fluoride in drinking water should be carefully monitored: a F^−^ concentration lower than 0.5 mg/L may lead to dental decay (Yuan et al., 2020); a high F^−^ concentration is linked with dental fluorosis (1.5–4.0 mg/L; Rwenyonyi et al., 1999) and skeletal fluorosis (>4 mg/L; He et al., 2020). Fluoride is toxic to microorganisms because it inhibits their phosphoryl-transfer enzymes (Breaker, 2012). With an acidic extracellular environment, unicellular organisms would take up the membrane permeable hydrofluoric acid (HF) and accumulate F^−^ in the cytoplasm (known as the weak acid accumulation effect) leading to F^−^-induced toxicity. Many microorganisms have developed fluoride resistance through the action of fluoride-exporting proteins to counter the weak acid accumulation effect. Recently, fluoride channels, CLC^F^ F^−^/H^+^ antiporters (Baker et al., 2012; Stockbridge et al., 2012; Brammer et al., 2014) and “Fluc” proteins (Ji et al., 2014; Stockbridge et al., 2014, 2015), have been found. These membrane proteins contain small pores to achieve the selectivity for fluoride, which is essential to the survival of unicellular organisms and eukaryotes under fluoride-containing environments.

However, the design of a fluoride selective transporter is still challenging, owing to the intrinsic nature of fluoride, in terms of small size and basic and high hydration energy. In fact, only a few examples of fluoride carriers are reported, including heavy pnictogenium cations (Park et al., 2019), phosphonium boranes (Park and Gabbai, 2020), and strapped calix[4]pyrroles (Clarke et al., 2016), but they are also active for chloride transport. In view of the small size of fluoride and the small pore in Fluc proteins that facilitates F^−^ selectivity, it is anticipated that creating a cyclic molecule with a small cavity could be a design strategy for fluoride selective transport. Meanwhile, secondary structures play a vital role in maintaining the shape and function of ion channel proteins (Ketchem et al., 1993). We therefore decided to introduce a small secondary structure, i.e., β-turn, into cyclic molecule to improve the lipophilic property of the transporter.

**Figure 1:**
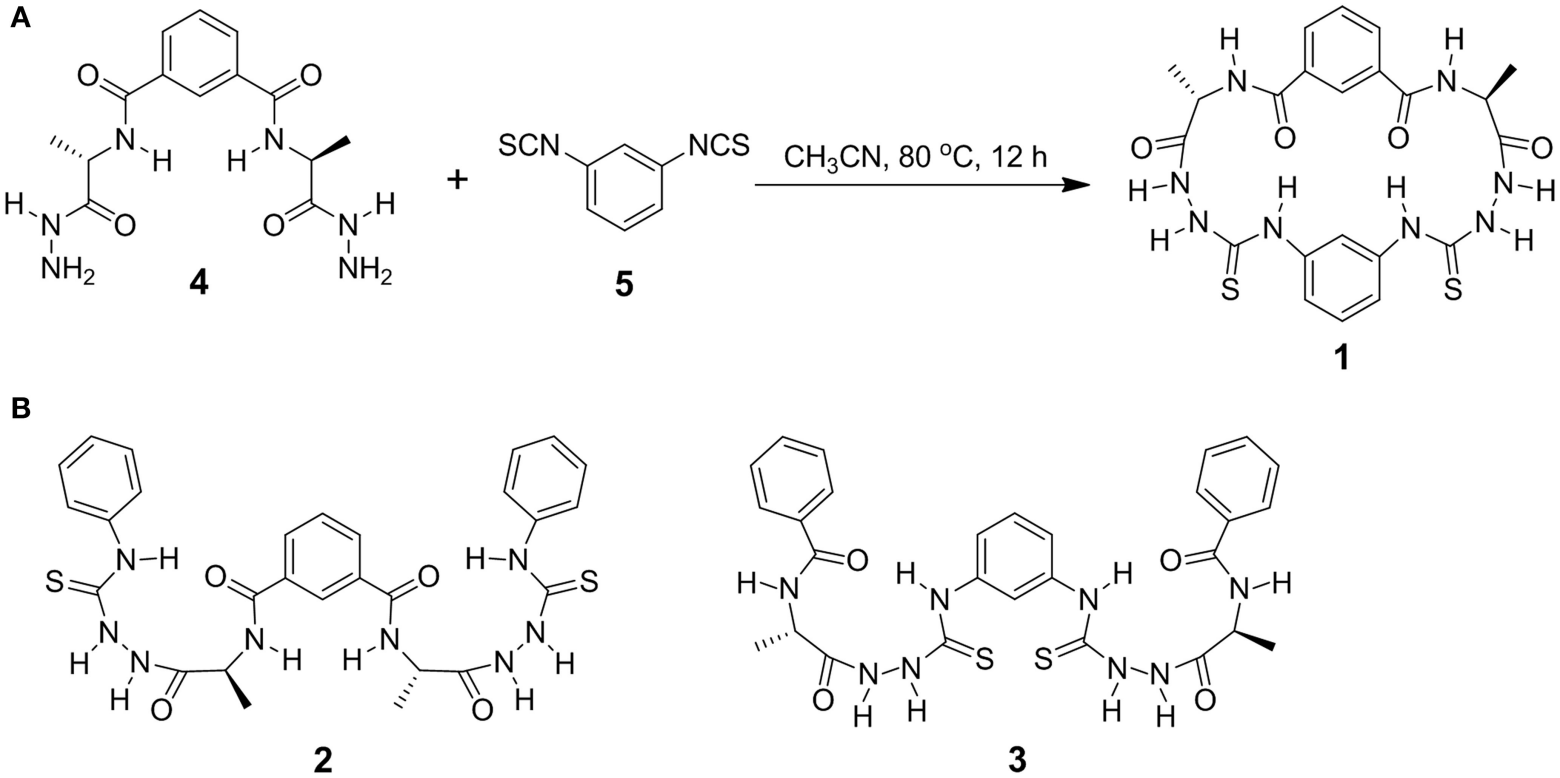
Synthesis of cyclic azapeptide **1 (A)** and molecule structures of acyclic counterparts **2** and **3 (B)**. All alanine residues are of L-configuration.

Herein, by employing alanine-based *N*-amidothiourea motif, a structural scaffold featuring β-turn structure (Yan et al., 2013, 2018, 2019; Cao et al., 2017), we design and synthesize a cyclic azapeptide **1** and two acyclic analogs **2** and **3** (Figure 1) for fluoride transmembrane transport. The facile synthesis of cyclic receptor **1** is facilitated by the *meta*-position of the two benzene linkers and the 2-folded β-turns, which offer a small confined cavity for fluoride binding, leading to good selectivity in fluoride transmembrane transport across lipid bilayers according to an electrogenic anion uniport mechanism. Acyclic control compounds **2** and **3** are able to bind fluoride via the thiourea moieties but show no fluoride transmembrane transport activity. From these results, it is believed that a cyclic host of small cavity with a conformation mimicking the secondary structure of proteins could be a key to designing effective and selective fluoride transporters.

## Results and Discussions

### Synthesis and Characterization

Equal molar bilateral alanine-based hydrazides **4** (0.67 g, 2 mmol) and 1,3-phenylene diisothiocyanate **5** (0.38 g, 2 mmol) were dissolved in 50 mL CH_3_CN and stirred at 80°C for 12 h (Figure 1A). Removing the solvent through evaporation in vacuum, the crude product showed a set of well-resolved ^1^H NMR signals (Supplementary Figure 1), indicating the formation of a main product with well-defined structure and of high purity and high yield. This product was suggested to be the cyclic azapeptide **1** according to the MALDI-TOF mass spectrum that shows a sharp peak of 528.03 (Supplementary Figure 2, calcd for [**1**+H]^+^ [C_22_H_24_N_9_O_4_S_2_]^+^: 529.14). After recrystallizing in CH_3_CN, pure product **1** of high isolated yield (72%) was obtained. Generally, the two-point reaction could result in diverse oligomers, polymers, and cyclic compounds, which have not been found during the synthesis of **1**. The *meta*-position of the two benzene linkers and the folded β-turn structures are expected to contribute to the accessible synthesis of **1**. Acyclic analogs **2** and **3** (Figure 1B) were synthesized according to the procedures described in Scheme S1 in Supplementary Material.

### β-Turn Structures

**Figure 2:**
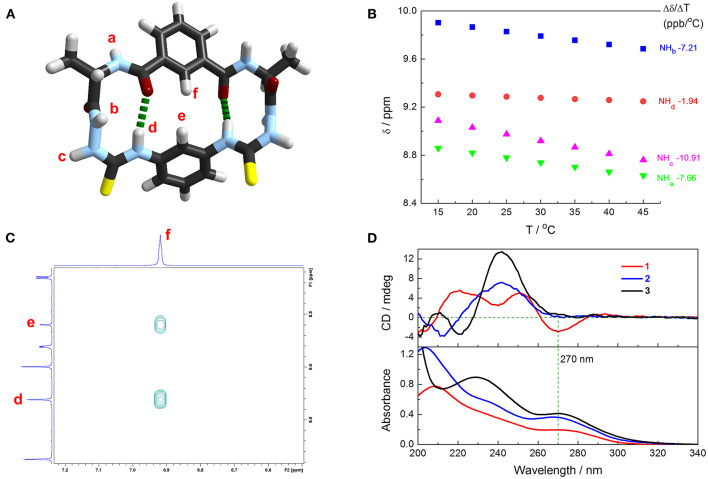
**(A)** Calculated structure of cyclic azapeptide **1** with two β-turns. Dashed green lines highlight the intramolecular hydrogen bonds of β-turn structures. Method for calculation: B3LYP DFT with the 6-311G** basis set. **(B)** Influence on –NH protons resonances of **1** in 90:10 (v/v) CD_3_CN/DMSO-*d*_6_ mixtures by temperatures (600 MHz) and the fitted temperature coefficients. [**1**] = 1 mM. **(C)** Partial 2D NOESY spectrum of **1** in 90:10 (v/v) CD_3_CN/DMSO-*d*_6_ mixtures. [**1**] = 1 mM. **(D)** Absorption and CD spectra of **1** (red line), **2** (blue line), and **3** (black line) in CH_3_CN. [**1**] = [**2**] = [**3**] = 20 μM.

β-Turn structure has been well-identified in alanine-based *N*-amidothiourea in our previous works. The calculated structure of cyclic azapeptide **1** features two β-turns as shown in Figure 2A. ^1^H NMR spectra of **1** were recorded in 90:10 (v/v) CD_3_CN–DMSO-*d*_6_ mixtures at variable temperatures (Supplementary Figure 3), from which the temperature coefficients of the chemical shift of –NH protons were obtained (Figure 2B). Different from –NH_a_ (−7.66 ppb/°C), –NH_b_ (−7.21 ppb/°C), and –NH_c_ (−10.91 ppb/°C), which have very negative values, a much more positive temperature coefficient of thioureido –NH_d_ (−1.94 ppb/°C) was observed, suggesting the protection of –NH_d_ by intramolecular interaction, ascribed to the intramolecular 10-membered ring hydrogen bonds that maintain the two β-turns in **1** (Figure 2A) (Cao et al., 2017; Yan et al., 2019; Zhang et al., 2020). The obvious NOE signals of H_e_-H_f_ and H_d_-H_f_ again support the folded β-turn structures in cyclic azapeptide **1** (Supplementary Figure 4 and Figure 2C).

Folded β-turn structures also exist in acyclic control compounds **2** and **3**, suggested by the DFT calculated structures (Supplementary Figure 5), as well as the temperature coefficients of the chemical shift of the –NH protons (Supplementary Figures 6–9). Given the more positive temperature coefficient of the –NH_d_ in **1** (-1.94 ppb/°C) than those in **2** and **3** (−2.65 and −2.64 ppb/°C, respectively, Table 1), it is believed that the intramolecular hydrogen bonds in **1** are more stable than those in **2** and **3**, owing to the rigid cyclic conformation that stabilizes the β-turn structures. Meanwhile, CD spectra in CH_3_CN show an obvious CD signal at 270 nm of **1** and weak signals for **2** and **3**, suggesting the more efficient intramolecular chirality transfer from chiral alanine residues to achiral phenylthiourea chromophore in **1**, deduced from the absorption spectra (Figure 2D). Meanwhile, the cyclic conformation also leads to higher lipophilicity of **1**, supported by their retention times in reverse-phase HPLC (Supplementary Table 1). This could be a potential advantage of **1** for anion transmembrane transport (Valkenier et al., 2014).

**Table 1:** Temperature coefficients of –NH protons' chemical shifts of **1**, **2**, and **3** in 90:10 (v/v) CD_3_CN/DMSO-*d*_6_ mixtures.

**Compound**	**1**	**2**	**3**
Temperature coefficient (ppb/°C) -NH_a_	−7.66	−8.97	−9.63
-NH_b_	−7.21	−8.24	−8.41
-NH_c_	−10.91	−8.52	−8.84
-NH_d_	−1.94	−2.65	−2.64

### Anion Binding

**Figure 3:**
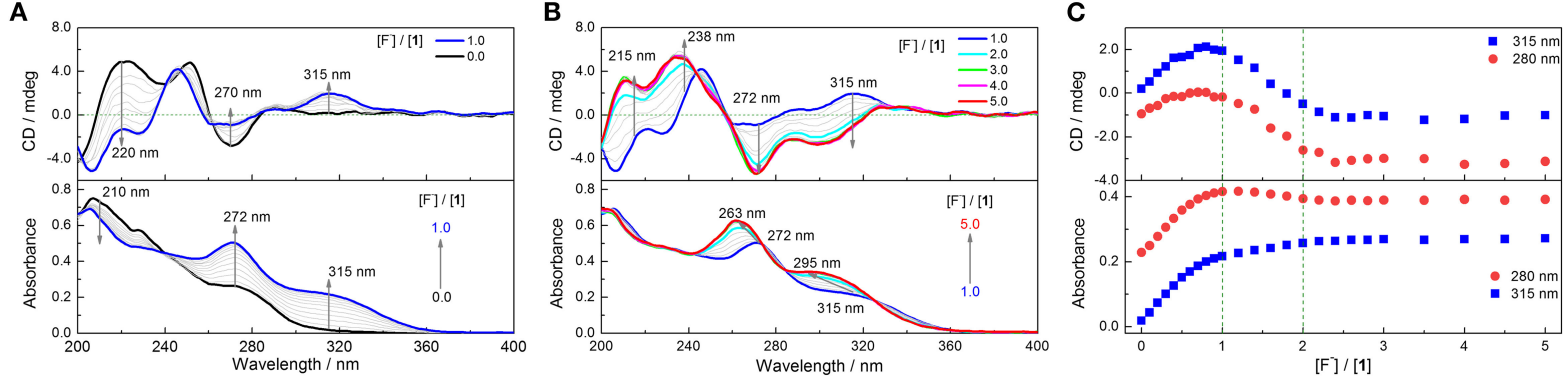
Absorption and CD spectra of **1** in CH_3_CN in the presence of F^−^ from 0 to 1.0 eq **(A)** and from 1.0 to 5.0 eq **(B)**. **(C)** Plots of absorbance and CD intensity at 280 and 315 nm vs. concentration of F^−^. [**1**] = 20 μM. F^−^ exists as the (*n*-Bu)_4_N^+^ salt.

The binding capacity of **1** toward halide anions in CH_3_CN was next examined. Despite no perceivable binding to Cl^−^, Br^−^, and I^−^ (Supplementary Figures 10–12), a substantial change in absorption and CD spectra of **1** was observed in the presence of F^−^ (Figure 3 and Supplementary Figure 13), undergoing multiple processes since the two binding groups, thiourea moieties, in **1** are in close proximity. Upon the addition of F^−^ from 0.0 to 1.0 eq, the absorbance at 272 nm that from the thiourea chromophore is increased, along with the development of a new band at 315 nm, which is assigned to the charge transfer band of the anion–thiourea complex (Figure 3A). Significantly, a new CD band develops at 315 nm, suggesting the occurrence of intermolecular chirality transfer. With increasing F^−^ concentration from 1.0 to 2.0 eq, the absorption at 272 nm blue shifts to 263 nm, while that at 315 nm shifts to 295 nm. While the CD signals at 272 and 315 nm become more negative, the positive signals at 238 and 215 nm are also enhanced, leading to bisignate and coupled Cotton effects at 272 and 238 nm (Figure 3B). From 2.0 to 5.0 eq F^−^, both absorption and CD spectra are changed slightly (Figure 3C). Thus, a final 1:2 binding complex is suggested for **1** in the presence of F^−^. The optimized structure of **1**·2F^−^ complex shows that one F^−^ interacts with **1** through three N–H···F^−^ hydrogen bonds in the cavity of each side, breaking the β-turn structures. Therefore, one **1** molecule binds two F^−^ ions through six hydrogen bonds (Supplementary Figure 14 and Supplementary Table 2), resulting in a high binding energy up to−158.12 kcal mol^−1^ or−79.06 kcal mol^−1^ per F^−^ ion. Moreover, the calculated CD spectrum performed by TD-DFT is similar to the experimental spectrum of **1** in the presence of 2.0 eq F^−^ (Supplementary Figure 15), indicating the reliability of the calculated binding model.

Acyclic compounds **2** and **3** can also bind with fluoride in CH_3_CN by employing two thiourea groups. In **2**, the two thiourea moieties are distant, so only one equal binding process is shown in the titration spectra, in which the absorbance at 292 nm can be assigned to the F^−^ binding complex of charge transfer transition (Supplementary Figure 16). Thereby, intermolecular chirality transfer is indicated by the emerged Cotton effects at 292 nm. Differently, the two thiourea groups in **3** are in close proximity, and the resultant binding complexes exhibit enhanced absorption and CD peaks at 266 nm, while the CD signals at 242 nm from the alanine residues are decreased (Supplementary Figure 17). The binding constants of acyclic **2** (*K* = 2.2 × 10^5^ M^−1^, Supplementary Figure 16) and **3** (K_11_ = 5.9 × 10^5^ M^−1^, K_12_ = 8.5 × 10^3^ M^−1^, Supplementary Figure 17) with F^−^ are fitted to be much lower than those of cyclic **1** with F^−^ (K_21_ = 1.7 × 10^5^ M^−1^, K_11_ = 1.9 × 10^8^ M^−1^, K_12_ = 8.6 × 10^5^ M^−1^, Supplementary Figure 13). No binding events are observed for **2** and **3** in the presence of Cl^−^, Br^−^, and I^−^ (Supplementary Figures 18–23).

### Osmotic Assay

**Figure 4:**
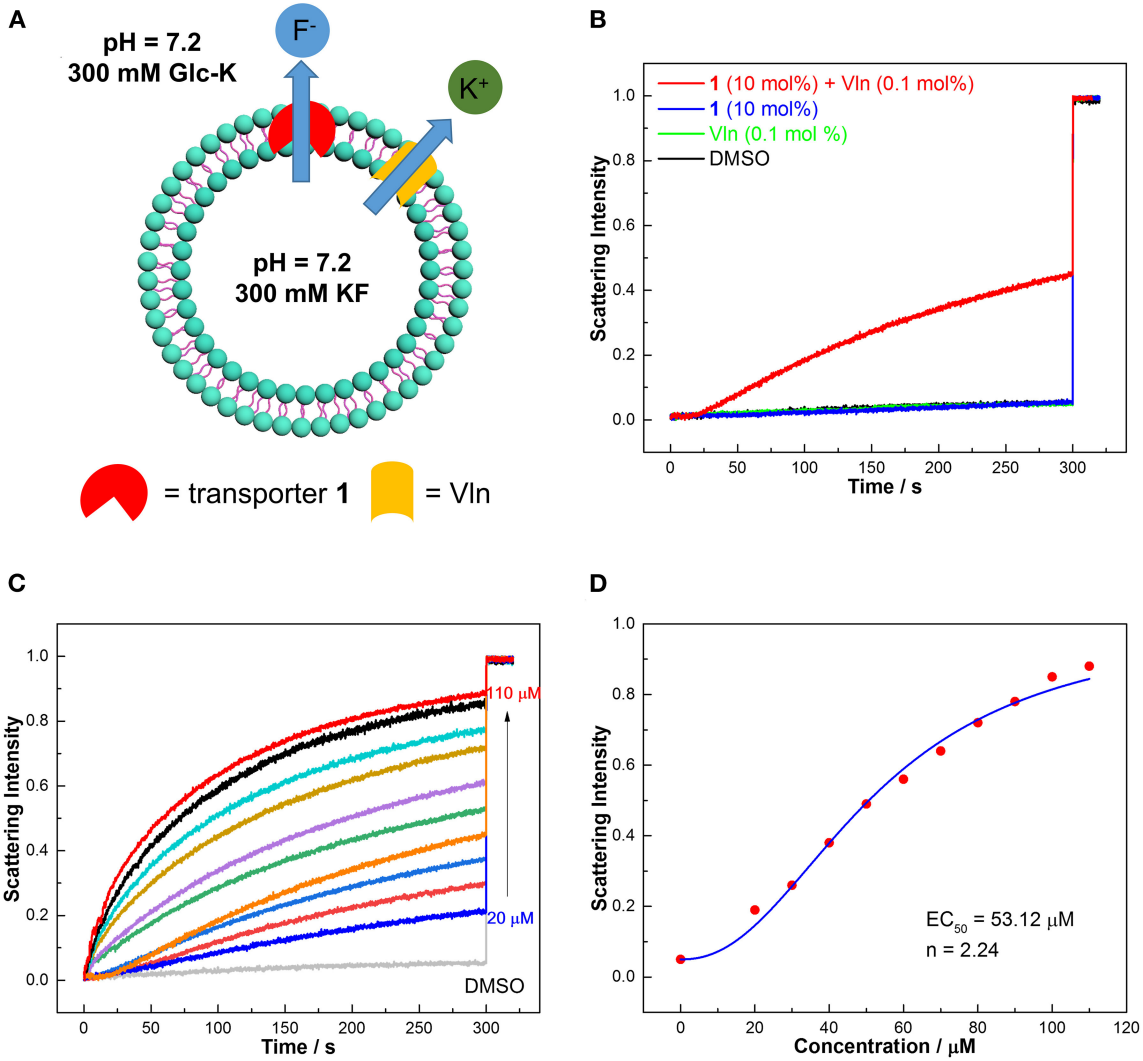
**(A)** Schematic representation of osmotic assay for fluoride transport by exerting a concentration gradient of fluoride. Inside LUVs: 100 mM KF, 10 mM HEPES, pH 7.2. Outside LUVs: 100 mM Glc-K, 10 mM HEPES, pH 7.2. **(B)** Normalized fluoride efflux obtained by the addition of compound **1** (50 μM, 10 mol%) in the presence and absence of Vln (0.5 μM, 0.1 mol%). At 300 s, FCCP (25 μM, 5 mol%) and Vln (0.5 μM, 0.1 mol%) (if not added previously) were added to obtain a light scattering intensity corresponding to 100% transport for calibration. **(C,D)** Hill plot analysis of fluoride efflux when **1** is coupled with Vln (0.5 μM, 0.1 mol%).

The selective binding of **1**–**3** to fluoride implies that they might be candidates for selective transport of fluoride. We thus studied the fluoride transport activities of **1–3** according to osmotic assay as shown in Figure 4A (Clarke et al., 2016). Large unilamellar vesicles (LUVs, 400 nm), composed of 1-palmitoyl-2-oleoyl-*sn*-glycero-3-phosphocholine (POPC), were loaded with a buffered KF solution (300 mM) and suspended in a buffered K^+^ gluconate solution (300 mM). Transporters were added in the presence and absence of valinomycin (Vln, 0.5 μM, 0.1 mol%) to initiate the transport process (Figure 4A). The large hydrophilic anion gluconate was used as the extravesicular anion, which rules out an anion exchange mechanism. Without Vln, a fluoride transporter will create a membrane potential under the concentration gradient of fluoride, preventing fluoride efflux. Then Vln, a natural potassium carrier, can dissipate the membrane potential by transporting potassium leading to observable fluoride efflux. The overall KF cotransport will give rise to an osmotic gradient and drive the efflux of water from vesicles, leading to vesicle shrinkage. This process can be monitored by a fluorometer as an increase of 90° light scattering that results from the vesicle shrinkage. In the absence of Vln, the compounds **1**–**3** show no response from osmotic assay (Supplementary Figure 24), suggesting that the transporters cannot facilitate K^+^/F^−^ symport or F^−^/gluconate antiport. Then Vln was added before the measurement began, and compound **1** leads to a significant increase in light scattering intensity, which was not observed for **2** and **3** (Figure 4B and Supplementary Figure 25), implying that transporter **1** is capable of transporting fluoride via an electrogenic anion uniport mechanism. The transport activity was quantified by employing transporter **1** at different concentrations (Figure 4C). Fitting the data with Hill equation, EC_50_ (the effective concentration to reach 50% of maximum transport at 300 s) and n (Hill coefficient) are obtained and shown in Figure 4D. The concentration of lipids in osmotic assay is 500 μM; the EC_50_ value (53.12 μM) corresponds to a transporter/lipid ratio of 10.62 mol%. The corresponding Hill coefficient n (2.24) is attributed to F^−^ being transported as a 2:1 (transporter:anion) complex, which presumably leads to more effective shielding of the negative charge of F^−^ compared with 1:1 and 1:2 complexes. Although acyclic counterparts **2** and **3** can bind fluoride as shown by absorption and CD titrations, they show no fluoride transport activity from the osmotic assay, even in the presence of Vln (Supplementary Figure 25), ascribed to their much lower lipophilicity (Supplementary Table 1), as well as lower F^−^ affinity.

### ISE Assay

**Figure 5:**
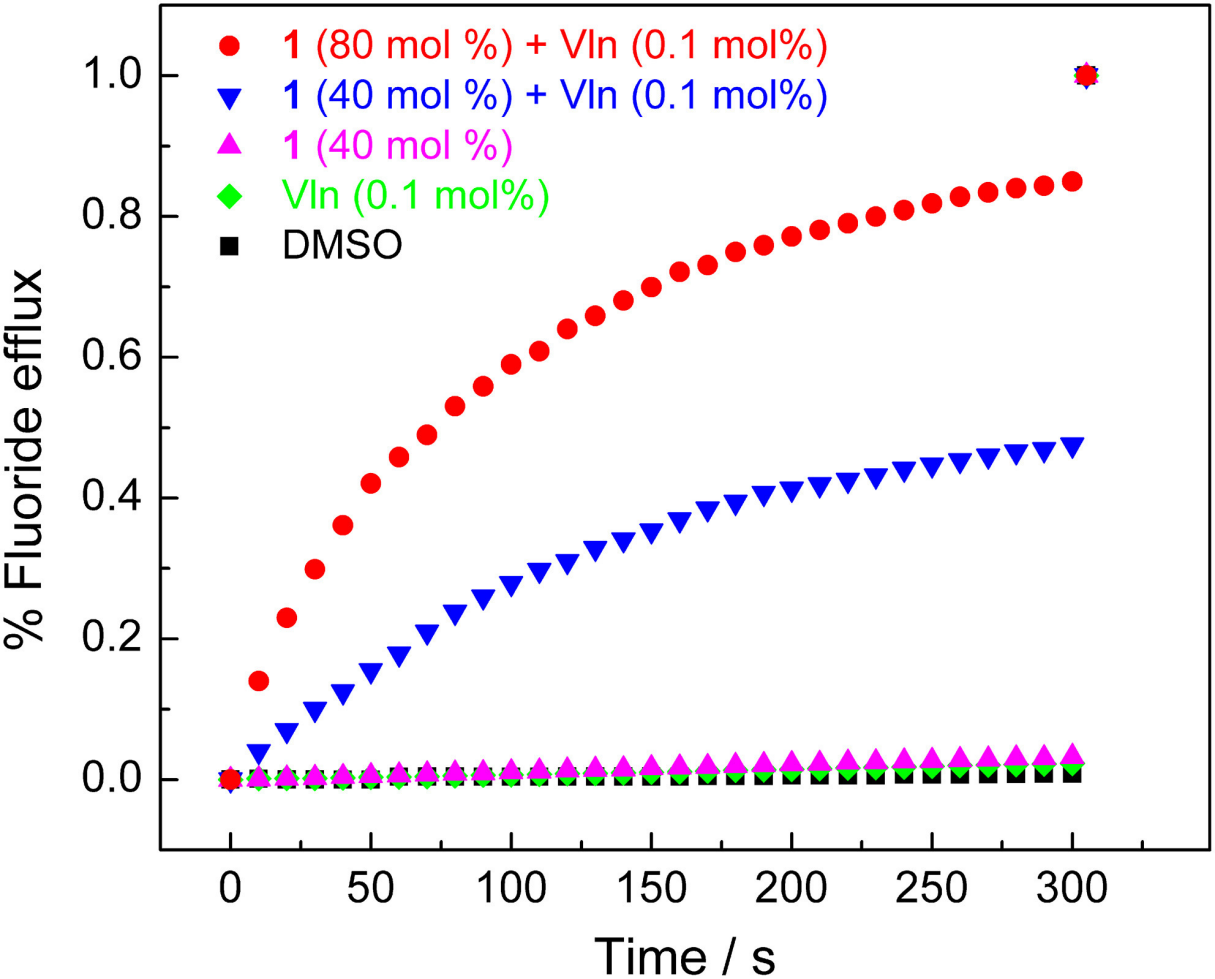
ISE assay for fluoride efflux obtained by addition of compound **1** (40 and 80 mol%) in the presence and absence of Vln (0.1 mol%).

Ion-selective electrode (ISE) assay was next applied to directly measure the fluoride transport and confirm the transport mechanism (Wu et al., 2016). POPC LUVs (mean diameter 200 nm) were prepared and used in this section. In the absence of Vln, negligible efflux of fluoride was detected, even when the concentration of transporter **1** is high up to 40 mol%. In the presence of Vln, the KF efflux could reach 47% in the presence of 40 mol% **1** and was increased to be 85% when the concentration of transporter **1** was up to 80 mol% (Figure 5). The transport activity of **1** depends on the presence or absence of Vln, confirming the electrogenic anion transport mechanism. Again, acyclic compounds **2**–**3** show negative response in the ISE assay (Supplementary Figure 26).

### Transport Mechanism Studies

**Figure 6:**
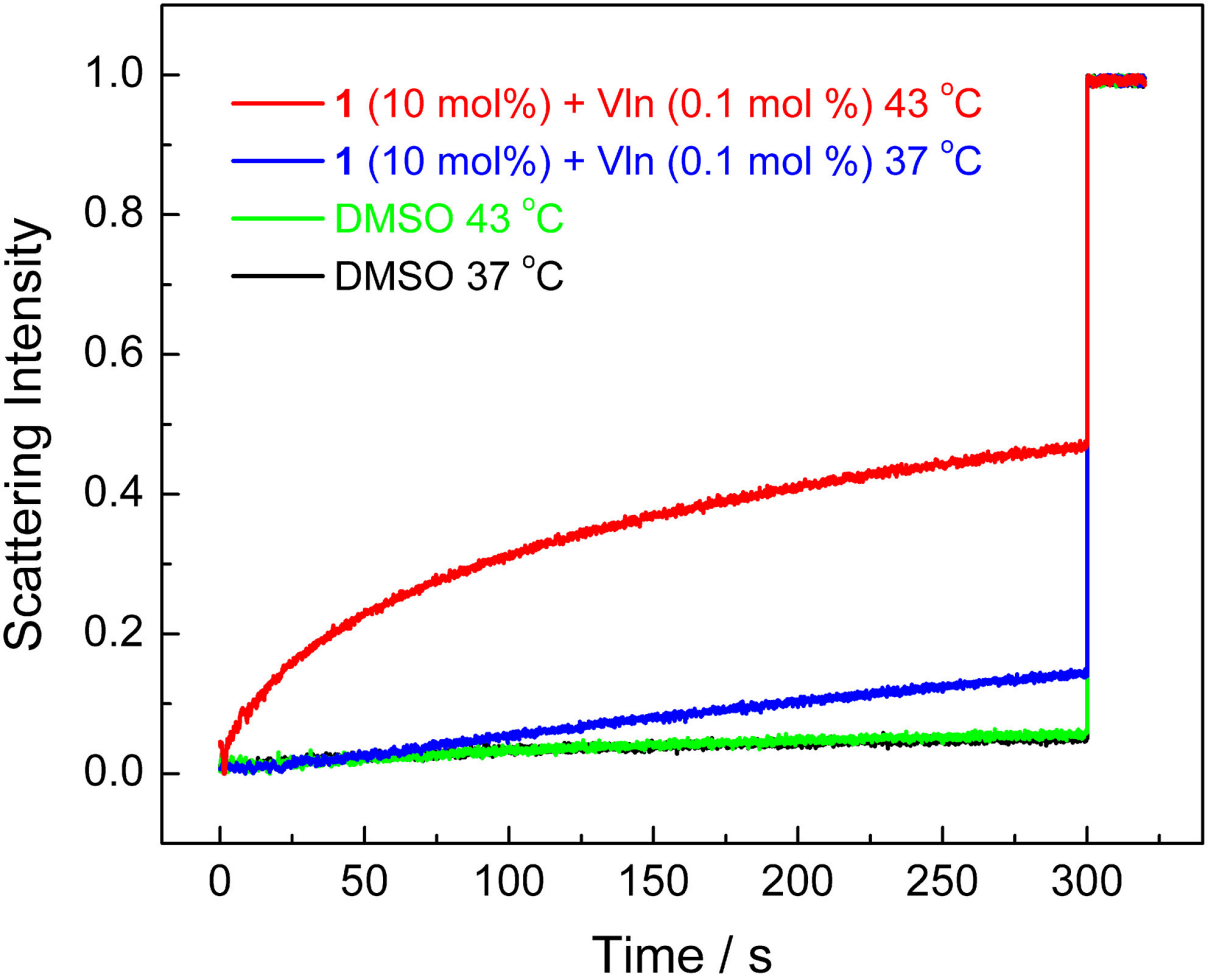
Normalized KF efflux quantified by lipids composed of DPPC under 37 and 43°C in the osmotic assay.

The transport mechanism (carrier or channel) was next investigated by employing lipids composed of dipalmitoylphosphatidylcholine (DPPC), which feature a phase transition at 41°C (Wu et al., 2015). The decreased fluidity of lipids below 41°C could suppress the transport activity of ion carriers but not ion channels. The amount of F^−^ transport of **1** at 300 s decreased from 47% at 43°C to 15% at 37°C in the osmotic assay (Figure 6), supporting the carrier transport mechanism.

It is noteworthy that HF is membrane permeable, and this could lead to an artifact in F^−^ transport studies when the transporter facilitates H^+^ (OH^−^) transport (Clarke et al., 2016). In the F^−^ transport artifact, the pH gradient generated by the HF efflux is dissipated by the H^+^ influx (or OH^−^ efflux) facilitated by the test transporter coupled to the K^+^ efflux facilitated by Vln, leading to the net KF efflux. Therefore, a H^+^ or OH^−^ transporter (e.g., FCCP) can generate a positive response in a F^−^ transport assay even if the transporter does not facilitate “true” F^−^ transport by reversibly binding F^−^ ions. KOAc-loaded vesicles were thus utilized to rule out H^+^ and OH^−^ transport, under which condition membrane-permeable acetic acid forms and would lead to the KOAc efflux in the presence of Vln and a H^+^ (OH^−^) transporter. Alternatively, acetate ion transport facilitated by a transporter would also lead to a response in the presence of Vln. If a transporter does not facilitate H^+^ (OH^−^) transport or acetate transport, acetate will stay inside the LUVs in the form of membrane-impermeable acetate ion. In our experiment, no detectable efflux of acetate was found (Supplementary Figure 27), confirming that transporter **1** is unable to transport H^+^ (OH^−^) or acetate. Thus, the KOAc-loaded osmotic assay, together with the KF-loaded osmotic assay, provides unambiguous evidence that cyclic compound **1** is capable of transporting fluoride via an electrogenic anion uniport mechanism. The integrity of the lipid bilayer can be evidenced by the calcein leakage assay (Wu et al., 2015). Calcein was encapsulated inside POPC LUVs; its fluorescence intensity was monitored to study whether pores are formed in lipids. Only negligible leakage of calcein occurred during the measurement (Supplementary Figure 28), confirming that cyclic compound **1** is functioning as a carrier rather than destroying the vesicle structure or forming large pores.

### Transport Selectivity of Fluoride Over Chloride

In view of the fact that the reported fluoride transporters are also active for chloride transport (Clarke et al., 2016; Park et al., 2019; Park and Gabbai, 2020), it is necessary to investigate the selectivity of fluoride over chloride. Hence, LUVs loaded with KCl (300 mM) were prepared to evaluate the transport activity toward chloride (Supplementary Figure 29A). Gratifyingly, no positive response could be observed in the Vln-coupled KCl efflux (Supplementary Figure 29B), suggesting the inactivity of **1–3** toward chloride transport, and thereby a transport selectivity of **1** toward fluoride over chloride. This highlights the importance of a small cyclic structure for selective ion transport.

## Conclusions

In summary, we developed a novel cyclic azapeptide, a peptide-based *N*-amidothiourea **1**, which can selectively bind and transport fluoride across lipid bilayers. NMR studies and DFT calculations verified the existence of β-turn structures in cyclic **1** and acyclic control compounds **2** and **3**, with the cyclic structure affording the strongest hydrogen bonding in β-turn structures. Osmotic and ISE assays were employed to explore the transport activities toward fluoride and chloride. We found that cyclic azapeptide **1** can act as a carrier to transport fluoride, operating via an electrogenic anion uniport mechanism. The cyclic structure with a confined cavity facilitates the encapsulation of fluoride, which is the key to the selectivity in anion binding and transport. These results illustrate the significance of encapsulation and offer instructions for design halide-selective anion receptors and transporters.

## Materials and Methods

### Materials

Isophthalic acid, benzoic acid, L-alanine, 1,3-phenylene diisothiocyanate, phenyl isothiocyanate, acetonitrile for reaction, and acetonitrile for spectroscopy were purchased from Sigma Aldrich Co. and used directly without purification. Acetonitrile-D_3_ and dimethyl sulfoxide-D_6_ were purchased from Cambridge Isotope Laboratories Inc. All other starting materials were obtained from Sinopharm Chemical Reagent Ltd.

### Instrumentation

^1^H and ^13^C NMR spectra were recorded on a Bruker AV600MHz or AV850MHz spectrometer. High-resolution mass spectra (HRMS) were acquired on a Bruker micro TOF-Q-II mass spectrometer. Circular dichroism (CD) spectra were recorded on Jasco J-1500. Absorption spectra were recorded on a Shimadzu UV-2700 UV-Vis spectrophotometer. Fluorescence spectra were obtained on a Horiba Fluorolog-3 spectrometer or a Hitachi F-7000 spectrometer. ISE assay was conducted by an Orion Dual Star.

### Synthetic Procedures for 1–3

Cyclic compound **1** and reference linear compound **2–3** were synthesized according to reported literatures with little modification (Yan et al., 2019). Isophthaloyl dichloride was synthesized by refluxing isophthalic acid (10.0 mmol) with SOCl_2_ (15.0 mmol) in CH_2_Cl_2_ (50.0 mL) overnight, evaporating solvent and unreacted SOCl_2_ on a rotary evaporator to obtain white solid without further purification. Isophthaloyl dichloride was mixed with **AOEt·HCl** (2.0 g, 13.0 mmol) and Et_3_N (3.0 mL) in CHCl_3_ (50.0 mL) and stirred overnight. The mixture was washed successively with **NH**_**3**_ · **H**_**2**_**O** (1%, 50.0 mL), HCl (1%, 50.0 mL), and saturated NaCl solutions (50.0 mL). Then pure product **6** (80% yield averagely) was obtained by drying the solution over anhydrous Na_2_SO_4_ and concentrating *in vacuo*. To synthesize product **4**, excess aqueous hydrazine (85%, 6.0 mL) was added to **6** in EtOH (50.0 mL) and refluxed the mixture for 24 h. The solvent was removed by evaporation to obtain a crude product and washed with CH_3_CN several times to afford pure white product **6** (65% yield averagely).

Linear compound **2** was synthesized by refluxing **4** (1.0 mmol) with an excess amount of **Ph(NCS)** (2.2 mmol) in CH_3_CN for 24 h. Pure product **2** (90% yield averagely) precipitated from the solution and could be used directly without further purification.

**8** was synthesized and purified under the similar way as **4**, then **3** can be acquired by refluxing excess **8** (2.2 mmol) with **5** (1.0 mmol) in CH3CN for 24 h. After filtrating, pure solid product **3** was obtained (90% yield averagely).

**1**: ^1^H NMR (600 MHz, DMSO-*d*_6_): δ (ppm) 10.44 (s, 2H), 9.81 (s, 2H), 9.28 (d, *J* = 5.3 Hz, 2H), 9.23 (s, 2H), 8.61 (s, 1H), 8.19 (dd, *J* = 7.8, 1.7 Hz, 2H), 7.86 (dd, *J* = 8.1, 1.9 Hz, 2H), 7.65 (t, *J* = 7.7 Hz, 1H), 7.36 (t, *J* = 8.1 Hz, 1H), 6.91 (t, *J* = 1.9 Hz, 1H), 4.32–4.25 (m, 2H), 1.38 (d, *J* = 7.1 Hz, 6H); ^13^C NMR (214 MHz, DMSO): δ (ppm) 180.98, 171.88, 167.21, 139.44, 133.88, 130.71, 128.77, 128.55, 127.48, 123.84, 120.30, 49.69, 16.75; HRMS (ESI): calcd for [C_22_H_24_N_8_O_4_S_2_Na]^+^: 551.1254, found: 551.1245.

**2**: ^1^H NMR (600 MHz, DMSO-*d*_6_): δ (ppm) 10.43 (s, 2H), 9.75 (s, 2H), 9.30 (s, 2H), 9.04 (s, 2H), 8.43 (s, 1H), 8.06 (d, *J* = 7.4 Hz, 2H), 7.65 (d, *J* = 25.5 Hz, 4H), 7.61 (t, *J* = 7.6 Hz, 1H), 7.33 (t, *J* = 7.3 Hz, 4H), 7.14 (t, *J* = 6.9 Hz, 2H), 4.40 (s, 2H), 1.43 (d, *J* = 6.3 Hz, 6H); ^13^C NMR (151 MHz, DMSO): δ (ppm) 180.74, 172.28, 167.48, 139.48, 134.19, 131.04, 128.74, 128.62, 127.69, 125.26, 124.62, 49.52, 17.19.; HRMS (ESI): calcd for [C_28_H_30_N_8_O_4_S_2_Na]^+^: 629.1724, found: 629.1726.

**3**: ^1^H NMR (600 MHz, DMSO-*d*_6_): δ (ppm); 10.43 (s, 2H), 9.76 (s, 2H), 9.38 (s, 2H), 8.92 (s, 2H), 8.07 (s, 1H), 7.93 (d, *J* = 7.3 Hz, 4H), 7.54 (t, *J* = 7.3 Hz, 2H), 7.49 (t, *J* = 7.4 Hz, 4H), 7.41 (d, *J* = 7.7 Hz, 2H), 7.32 (t, *J* = 8.0 Hz, 1H), 4.34 (s, 2H), 1.41 (d, *J* = 6.7 Hz, 6H); ^13^C NMR (214 MHz, DMSO): δ (ppm) 180.71, 172.37, 167.97, 139.38, 133.66, 132.09, 128.75, 128.21, 127.94, 121.84, 121.68, 49.58, 17.06; HRMS (ESI): calcd for [C_28_H_30_N_8_O_4_S_2_Na]^+^: 629.1724, found: 551. 629.1713.

### Osmotic Response Assay

The large unilamellar vesicles (LUVs, mean diameter 400 nm) of POPC were prepared by freeze/thaw cycles; 30.4 mg POPC dissolved in CHCl_3_ (50 mL) was slowly evaporated using a rotary evaporator and dried under high vacuum at room temperature for 4 h. The obtained lipid film was rehydrated by mixing with 4 mL saline solution (300 mM KF, 10 mM HEPES, pH 7.2) and vortexing for 2 min. Then the mixture was subjected to 10 freeze–thaw cycles and extruded through a 0.45-μm polycarbonate membrane for at least five times. Then the suspension (10 mM LUVs) can be used without further treatment.

In each run, 100 μL lipids stock (300 mM KF, 10 mM HEPES, pH 7.2, 10 mM LUVs) was added to 1.99 mL external solution (300 mM Glc-K, 10 mM HEPES, pH 7.2, 10 mM LUVs). DMSO (40 μL) solutions of transporters or DMSO (40 μL) was added at time 0. The light scattering intensity at 600 nm was monitored by a fluorimeter (λ_ex_ = 600 nm, λ_em_ = 600 nm). In this section, none of the three compounds show any response in osmotic assay, so valinomycin (Vln, 0.5 μM, 0.1 mol%) was added to accelerate the outflow of K^+^. If the outflow of F^−^ accompanied the outflow of K^+^, an osmotic gradient will be formed, causing vesicle shrinkage and increasing the amount of 90° light scattering. At 300 s, FCCP (25 μM, 5 mol%) and Vln (0.5 μM, 0.1 mol%) (if not added previously) were added to accelerate the transport process to the end for calibration. The fractional light scattering intensity (*I*_*f*_) was calculated based on the following equation:

$$\begin{array}{l} I_{f}=\frac{R_{t}-R_{0}}{R_{f}-R_{0}}\times 100\% \end{array}$$

In the equation, *R*_*t*_ is the light scattering intensity at time t, *R*_*f*_ is the final light scattering intensity obtained by the addition of FCCP and Vln, and *R*_0_ is the light scattering intensity at time 0. Different concentrations of the transporter molecule were added to obtain a series of fractional light scattering intensity (*I*_*f*_). Fitting *I*_*f*_ vs. molecule concentration was performed using the following equation:

$$\begin{array}{l} y=y_{0}+\left(y_{\text{max}}-y_{0}\right)\frac{x^{n}}{K+x^{n}} \end{array}$$

In the equation, *y* is the value of *I*_*f*_ corresponding to the carrier molecule loaded at concentration *x, y*_0_ is the *I*_*f*_ value measured at no compound added, *y*_*max*_ is the maximum *I*_*f*_ value, *n* is the Hill coefficient, and *K* is the EC_50_ value.

### ISE Assay

The large unilamellar vesicles (LUVs, mean diameter 200 nm) of POPC were prepared by freeze/thaw cycles; 50 mg POPC dissolved in CHCl_3_ (50 mL) was slowly evaporated using a rotary evaporator and dried under high vacuum at room temperature for 4 h. The obtained lipid film was rehydrated by mixing with a 5 mL saline solution (300 mM KF, 10 mM HEPES, pH 7.2) and vortexing for 2 min. Then the mixture was subjected to 10 freeze–thaw cycles and extruded through a 0.22-μm polycarbonate membrane for at least five times. The obtained lipid suspension (3.8 mL, 0.05 mmol) was transferred to size exclusion chromatography (stationary phase Sephadex G-50; mobile phase 300 mM Glc-K, 10 mM HEPES, pH 7.2) and diluted with the mobile phase to acquire 10 mL of 5 mM lipids stock solution.

In the F^−^ transport study, 1 mL of the lipid stock solution was added to 4 mL Glc-K (300 mM buffered to pH 7.2 using 10 mM HEPES) to generate a solution with a concentration gradient. As the former osmotic assay demonstrates that the transporter may work in an electrogenic way, Vln was added before the measurement. Then DMSO (40 μL) solutions of transporters or DMSO (40 μL) was added at time 0. The fluoride efflux was monitored by a fluoride selective electrode. At 300 s, Triton X-100 [100 μL, 20% (v/v)] was added as a detergent to lysate the LUVs for calibration.

### Ion Transport Mechanism Study by DPPC Experiments

DPPC LUVs (mean diameter 400 nm) were prepared as follows. A chloroform solution (30.4 mg DPPC) was evaporated slowly by a rotary evaporator and dried under high vacuum at room temperature for 4 h. After drying, the lipid film was rehydrated by vortexing with a 4 mL saline solution (300 mM KF, 10 mM HEPES, pH 7.2) and vortexing for 2 min at 55°C. The mixture was subjected to 10 freeze–thaw cycles (water bath was maintained at 55°C) and extruded through a 0.45-μm polycarbonate membrane for at least five times at 55°C. Then the suspension (10 mM LUVs) can be used without further treatment.

The transport of F^−^ was performed by the same way as above-mentioned. The lipid solution was stirred and thermostated in a polystyrene cuvette at 37 or 43°C.

### Ion Transport Mechanism by Calcein Leakage Assay

POPC LUVs (mean diameter 200 nm) were prepared as follows. A chloroform solution (10 mg POPC) was evaporated slowly by a rotary evaporator and dried under high vacuum at room temperature for 4 h. After drying, the lipid film was rehydrated by vortexing with a 1 mL calcein solution (100 mM NaCl, 10 mM HEPES, 100 mM calcein, pH 7.0) and vortexing for 2 min. The mixture was subjected to 10 freeze–thaw cycles and extruded through a 0.22-μm polycarbonate membrane for at least five times. The unentrapped calcein was separated by size exclusion chromatography (stationary phase Sephadex G-50; mobile phase 100 mM NaCl, 10 mM HEPES, pH 7.0) and diluted with the mobile phase to acquire 5 mL of 2 mM lipid stock solution.

For each run, 0.1 mL of the lipid stock solution was added to a 1.88 mL buffer solution to a final lipid concentration of 100 μM. DMSO (20 μL) solutions of transporters or DMSO (20 μL) was added at time 0. The fluorescence emission at time t (λ_ex_ = 495 nm, λ_em_ = 515 nm) was recorded over 30 min. At 30 min, Triton X-100 [10 μL, 20% (v/v)] was added as a detergent to lysate the LUVs for calibration. The fractional fluorescence intensity (*I*_*f*_) was calculated based on the following equation:

$$\begin{array}{l} I_{f}=\frac{R_{t}-R_{0}}{R_{f}-R_{0}}\times 100\% \end{array}$$

In the equation, *R*_*t*_ is the fluorescence intensity at time t, *R*_*f*_ is the final fluorescence intensity obtained by the addition of detergent, and *R*_0_ is the fluorescence intensity at time 0.

## Data Availability Statement

The original contributions presented in the study are included in the article/Supplementary Materials, further inquiries can be directed to the corresponding author/s.

## Author Contributions

XY and Y-BJ contributed to the conception and design of the study. ZZ and MZ finished the synthesis and transmembrane experiments. Absorption and CD spectra were collected by BT. PW and YZ carried out DFT calculations involved in this work. ZL contributed to the molecular design. All authors contributed to the article and approved the submitted version.

## Conflict of Interest

The authors declare that the research was conducted in the absence of any commercial or financial relationships that could be construed as a potential conflict of interest.
