# Supplementary material for: Transmembrane Fluoride Transport by a Cyclic Azapeptide With Two β-Turns
Source: Front Chem. 2021 Jan 12;8:621323. doi: 10.3389/fchem.2020.621323 (PMC7835674; doi:10.3389/fchem.2020.621323)
Supplement: Supplementary file 1 [file Data_Sheet_1.DOCX]

Supplementary Material


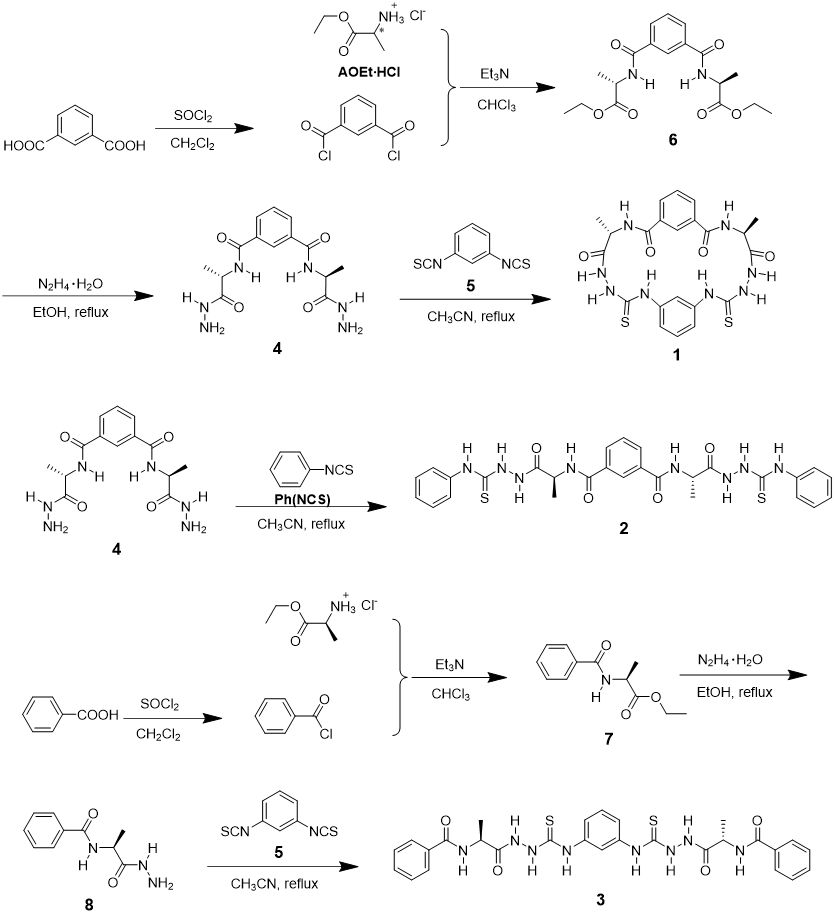


**Supplementary Scheme S1.** General procedures for the synthesis of **1-3**.


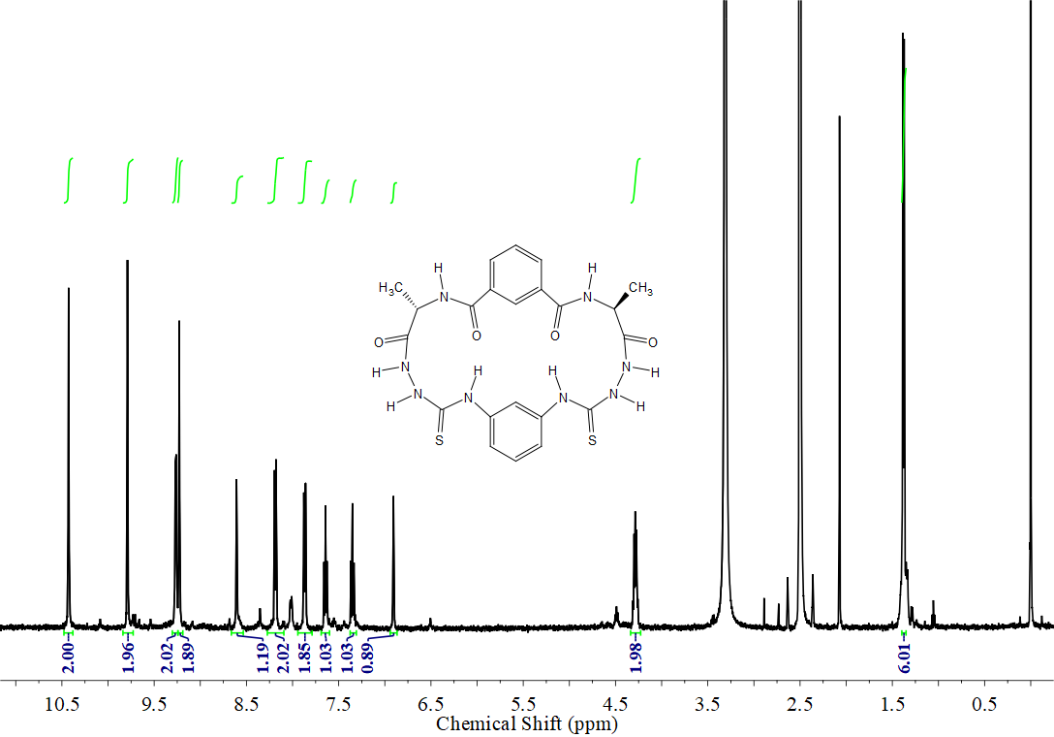


**Supplementary Figure S1.** ^1^H NMR spectrum (DMSO-*d*_6_) of crude product from the reaction of bilateral alanine-based hydrazides **4** with 1,3-phenylene diisothiocyanate **5**.


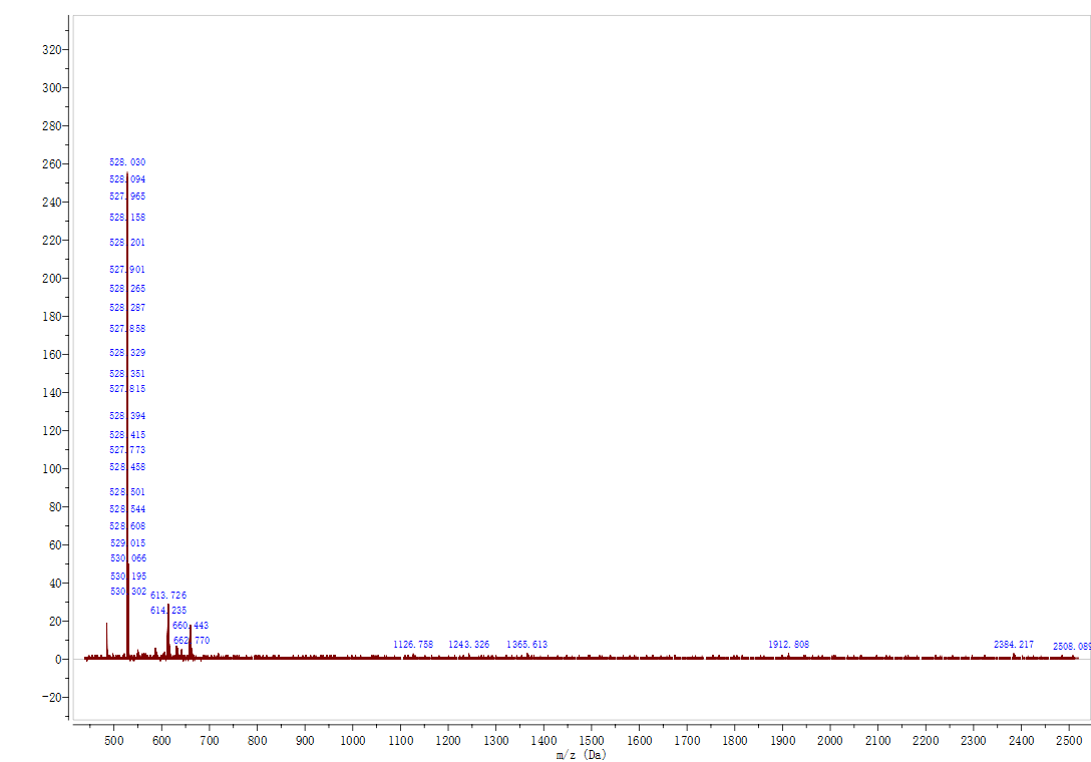


**Supplementary Figure S2.** MALDI-TOF mass spectrum of crude product from the reaction of bilateral alanine-based hydrazides **4** with 1,3-phenylene diisothiocyanate **5**.


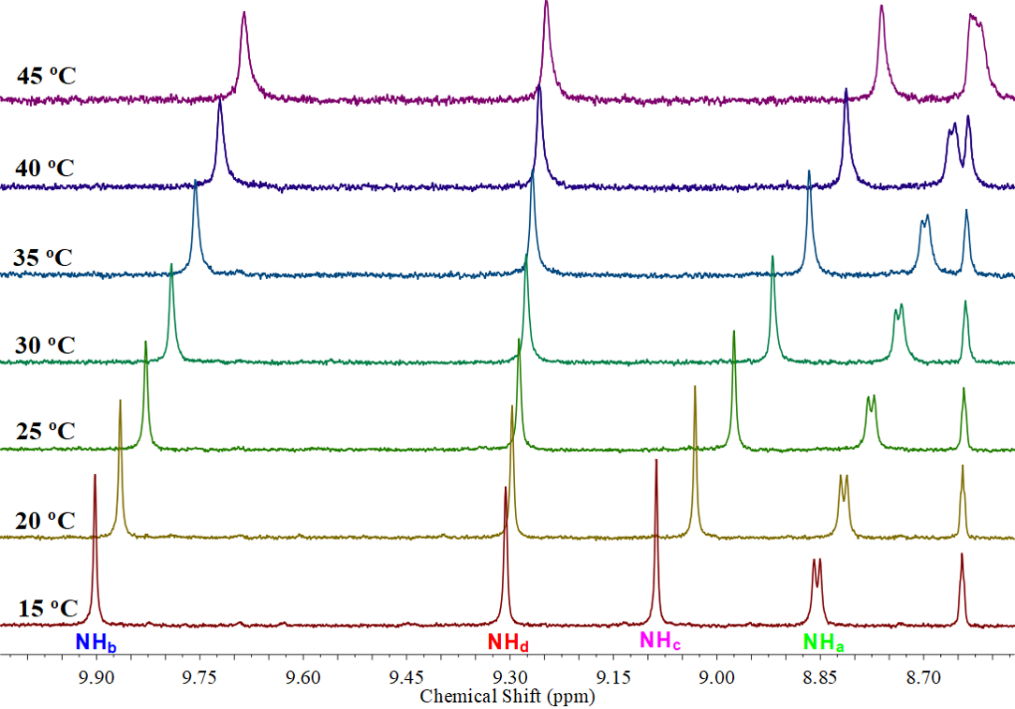


**Supplementary Figure S3.** Temperature-dependent partial ^1^H NMR spectra of –NH protons of **1** (600 MHz) in 90:10 (v/v) CD_3_CN/DMSO-*d_6_* mixtures. [**1**] = 1 mM.


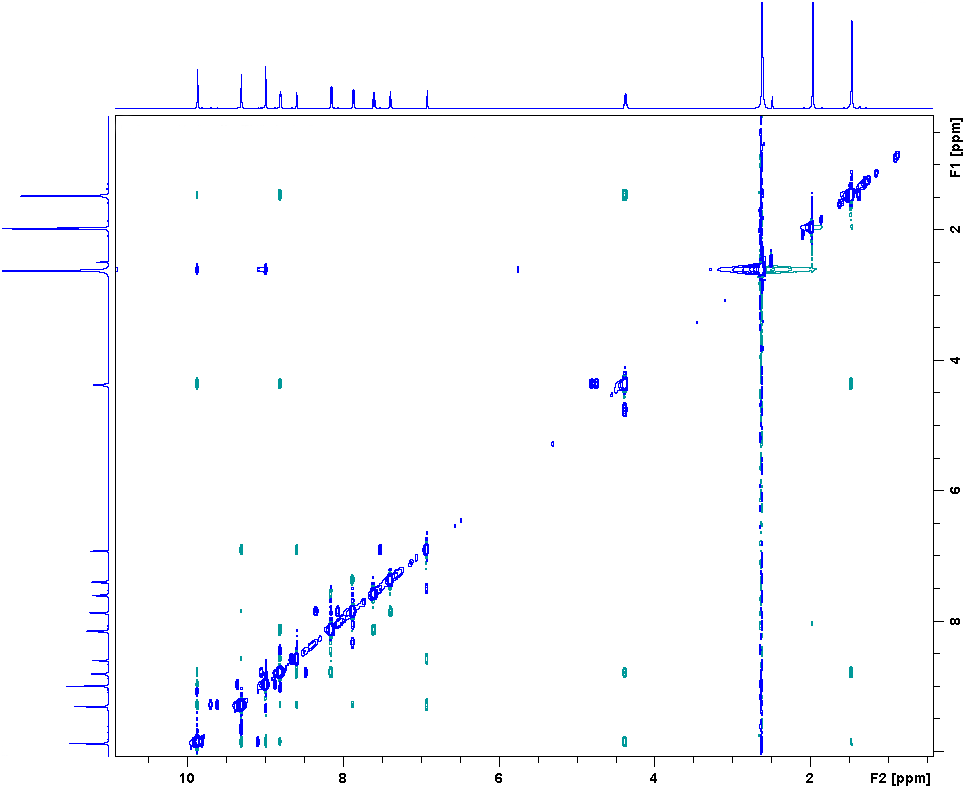


**Supplementary Figure S4.** 2D NOESY spectrum of **1** (600 MHz) in 90:10 (v/v) CD_3_CN/DMSO-*d_6_* mixtures. [**1**] = 1 mM.


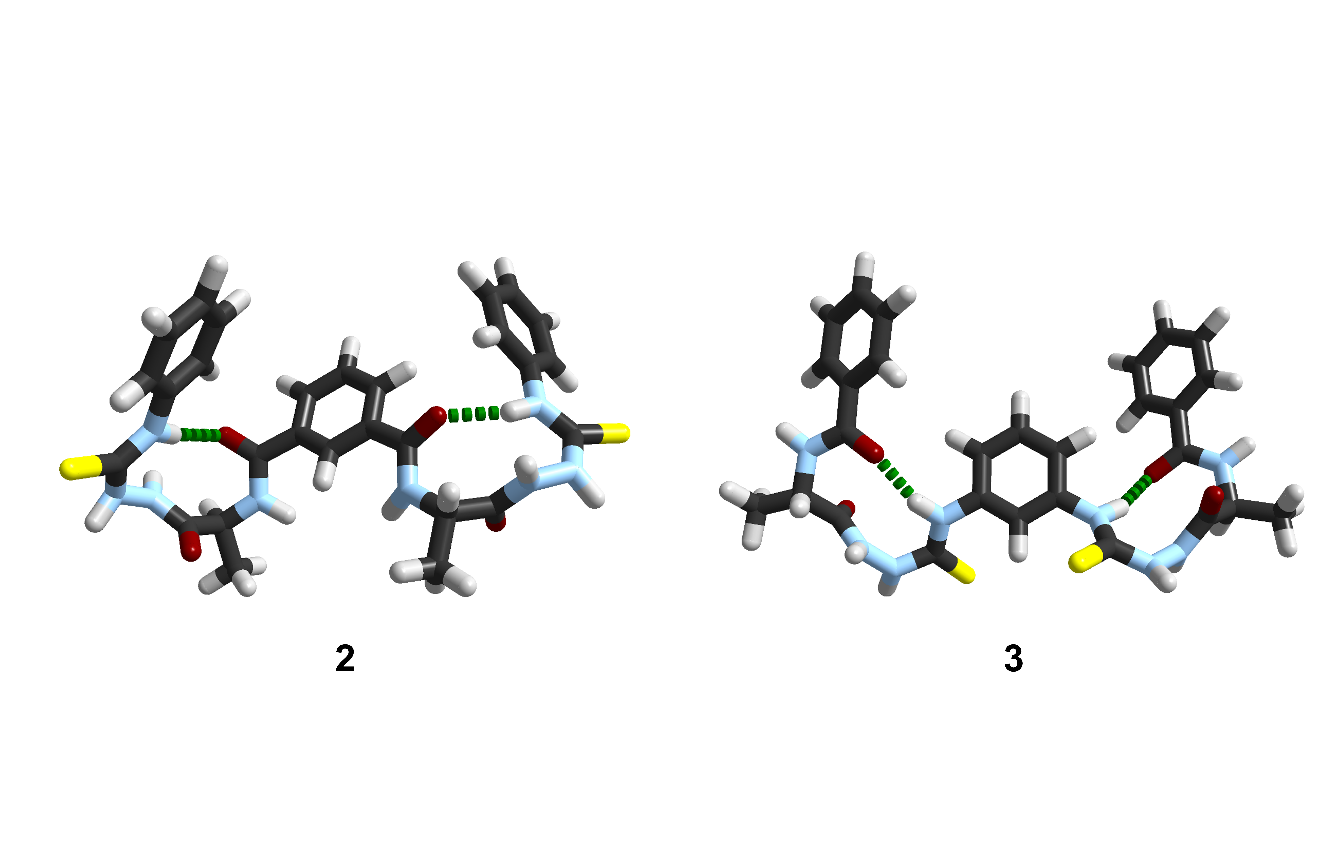


**Supplementary Figure S5.** Calculated structures of **2** and **3** with two β-turns. Dashed green lines highlight the intramolecular hydrogen bonds of β-turn structures. Method for calculation: B3LYP DFT with the 6-311G** basis set.


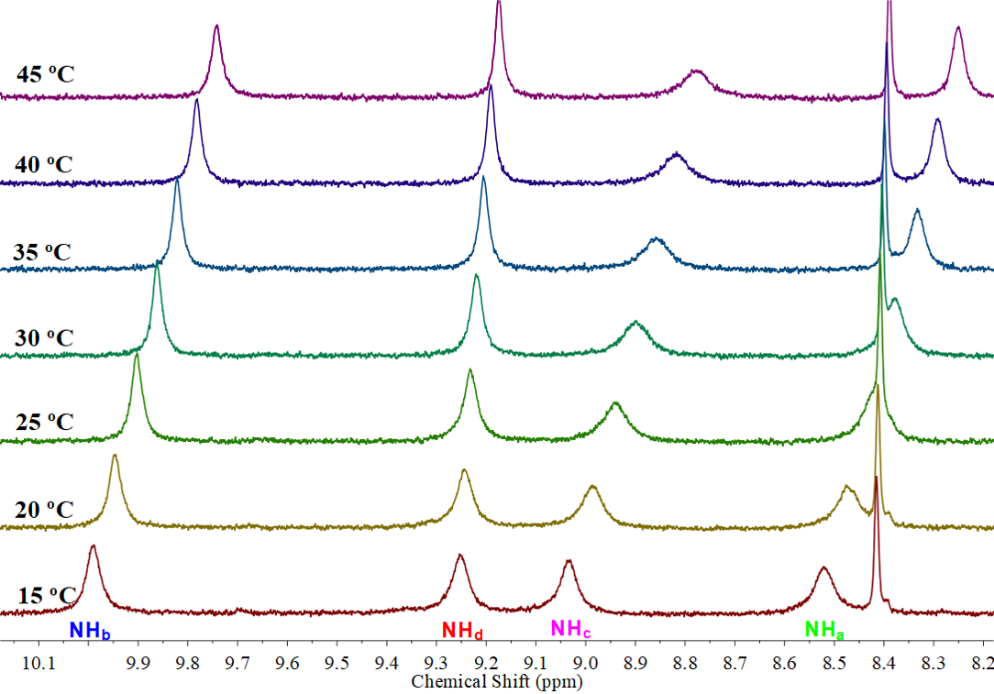


**Supplementary Figure S6.** Temperature-dependent partial ^1^H NMR spectra of –NH protons of **2** (600 MHz) in 90:10 (v/v) CD_3_CN/DMSO-*d_6_* mixtures. [**2**] = 1 mM.

**
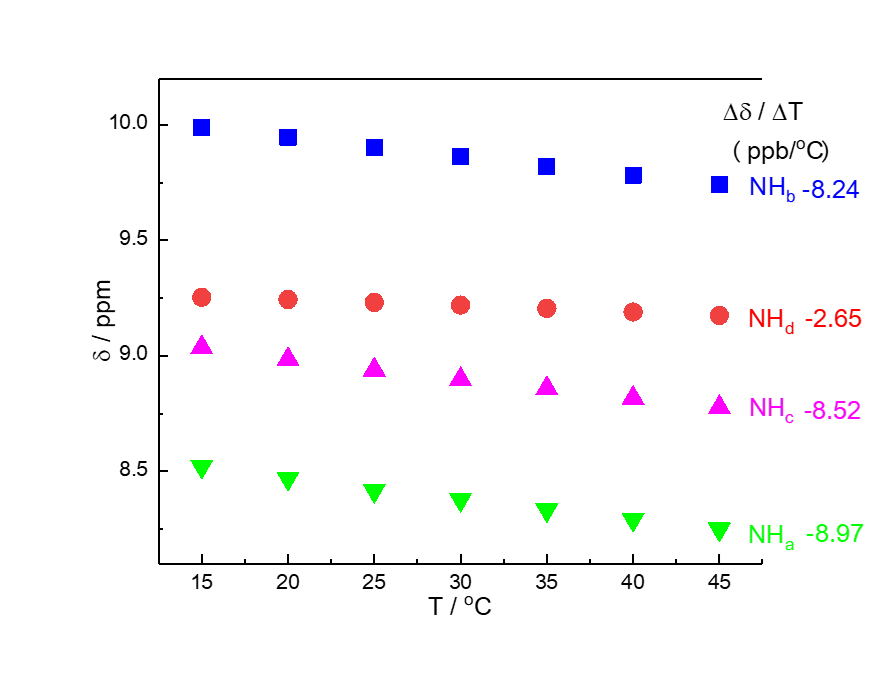
**

**Supplementary Figure S7.** Influence on –NH protons resonances of **2** in 90:10 (v/v) CD_3_CN/DMSO-*d_6_* mixtures by temperatures (600 MHz) and the fitted temperature coefficients. [**2**] = 1 mM.


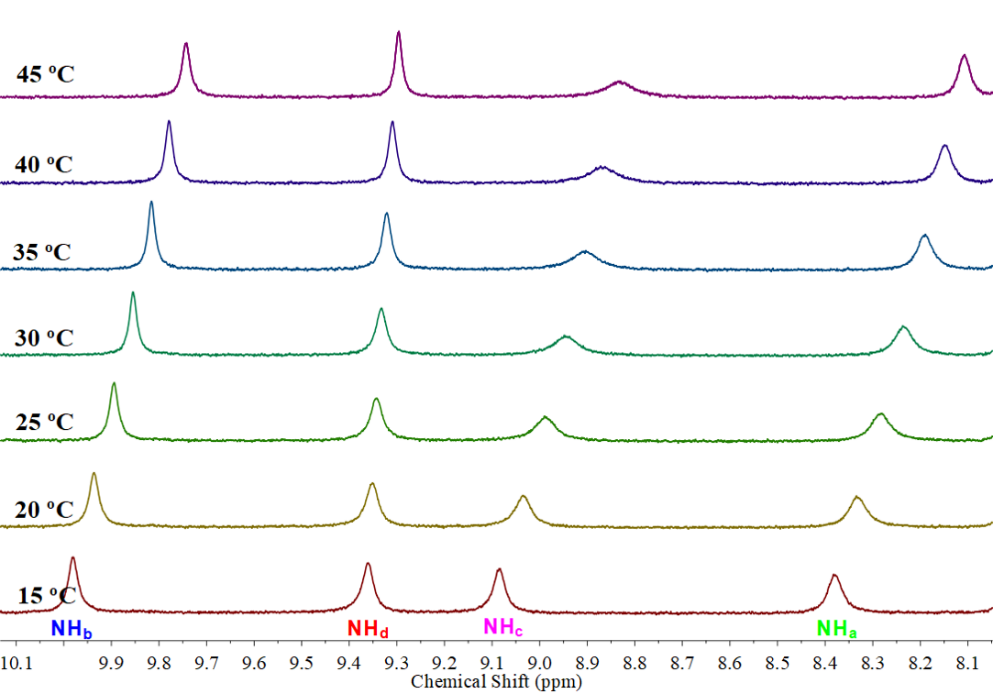


**Supplementary Figure S8.** Temperature-dependent partial ^1^H NMR spectra of –NH protons of **3** (600 MHz) in 90:10 (v/v) CD_3_CN/DMSO-*d_6_* mixtures. [**3**] = 1 mM.

**
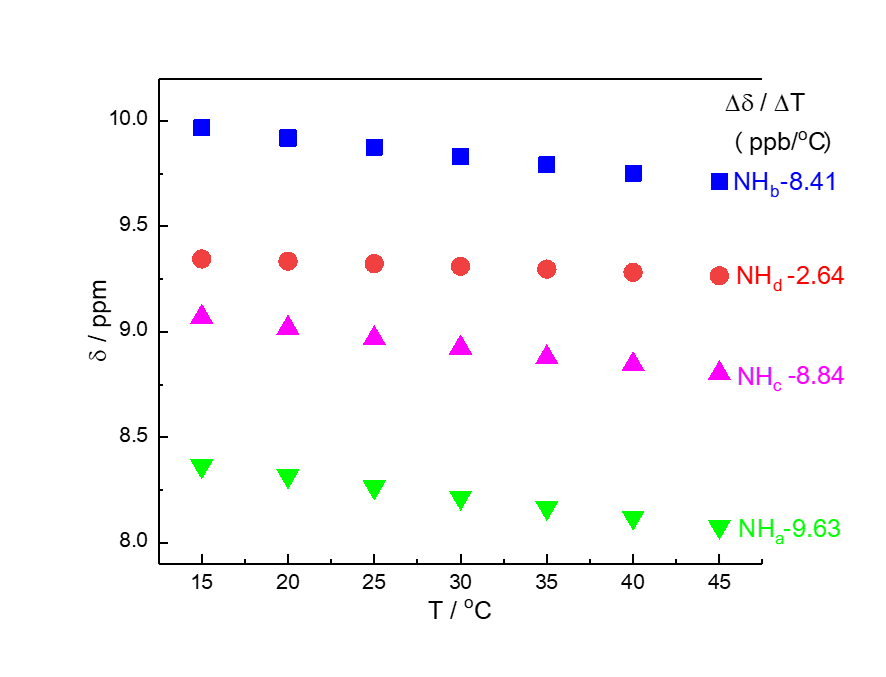
**

**Supplementary Figure S9.** Influence on –NH protons resonances of **3** in 90:10 (v/v) CD_3_CN/DMSO-*d_6_* mixtures by temperatures (600 MHz) and the fitted temperature coefficients. [**3**] = 1 mM.

**Supplementary Table S1.** Retention times of **1**, **2** and **3** in reverse-phase HPLC.*^a^*

| Compound | Retention time |
| --- | --- |
| **1** | 61.5 min |
| **2** | 38.8 min |
| **3** | 39.0 min |

*^a^* The relative lipophilicity of **1**, **2** and **3** can be estimated by comparing their retention times in reverse-phase HPLC experiments. The most retained compound of the most lipophilicity. HPLC conditions: HPLC column C18 (250 × 4.6 mm), injection volume 10 μL, eluent acetonitrile/water (0-5 min: 50/50, v/v; 5-70 min: gradient elution, 99/1, v/v), flow 1 mL/min, room temperature, λ = 270 nm.


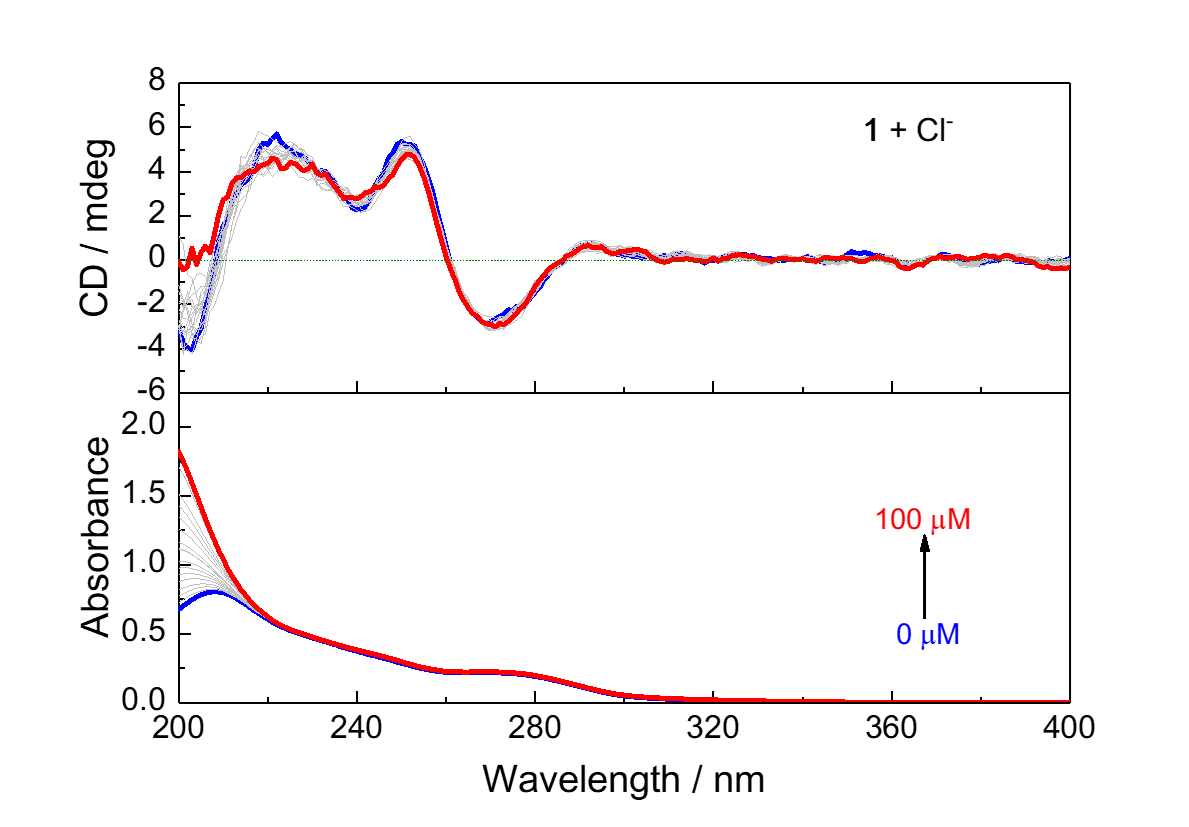


**Supplementary Figure S10.** Absorption and CD spectra of **1** in CH_3_CN in the presence of Cl^-^. [**1**] = 20 μM, [Cl^-^] = 0 to 100 μM. Cl^-^ exists as the (*n*-Bu)_4_N^+^ salt. The increased absorbance range from 200 to 220 nm comes from the absorption of Cl^-^.

**
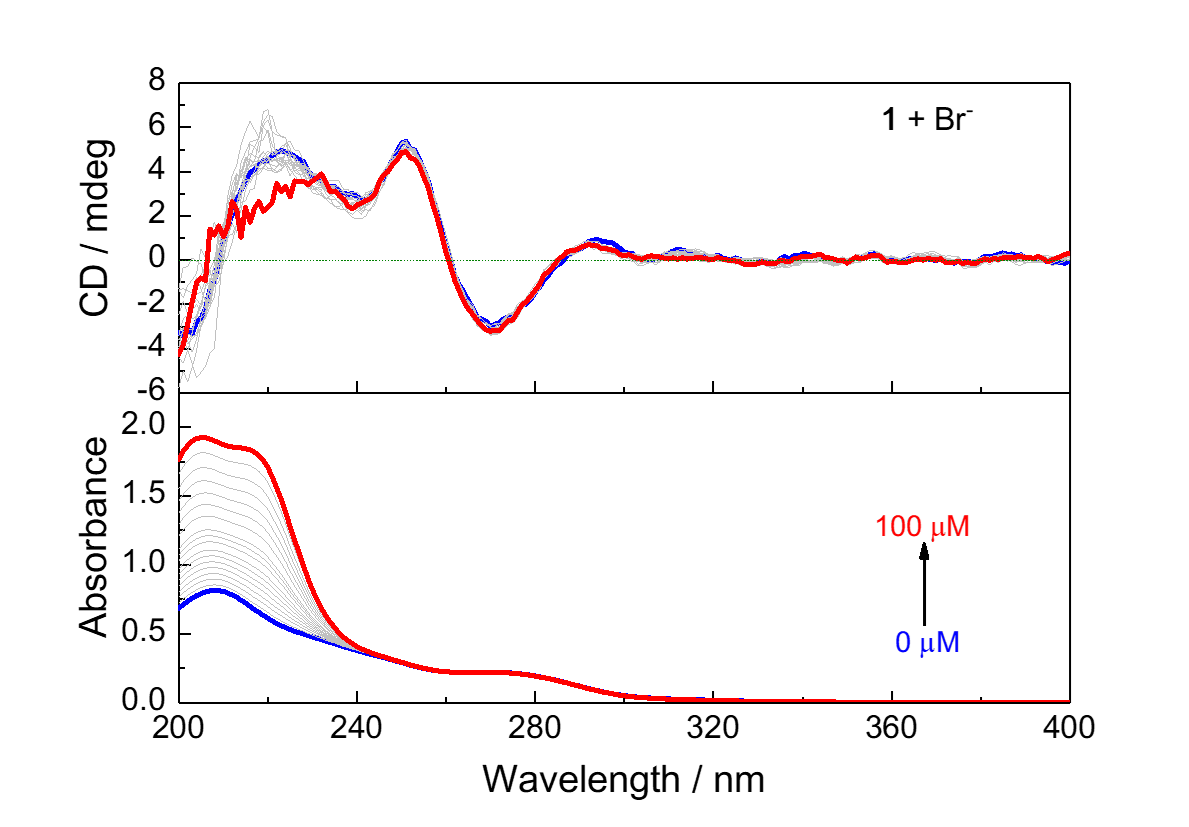
**

**Supplementary Figure S11.** Absorption and CD spectra of **1** in CH_3_CN in the presence of Br^-^. [**1**] = 20 μM, [Br^-^] = 0 to 100 μM. Br^-^ exists as the (*n*-Bu)_4_N^+^ salt. The increased absorbance range from 200 to 240 nm comes from the absorption of Br^-^.


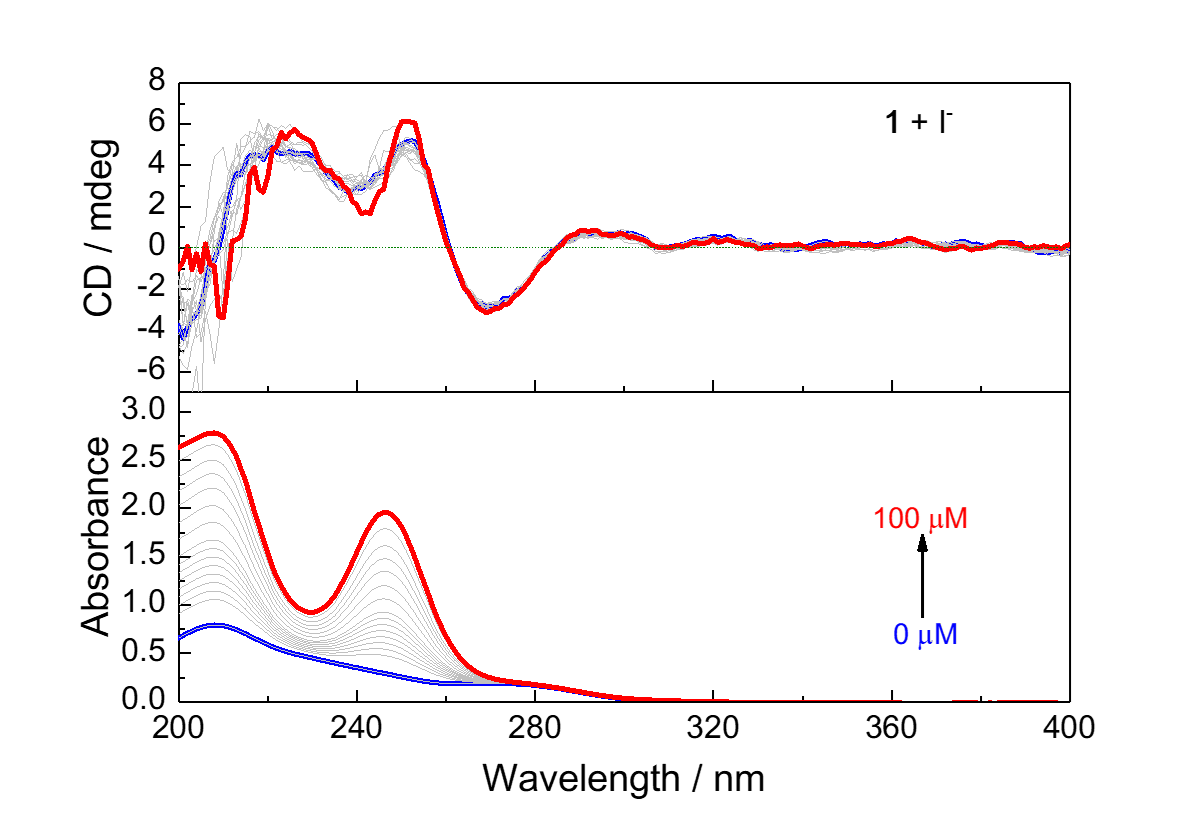


**Supplementary Figure S12.** Absorption and CD spectra of **1** in CH_3_CN in the presence of I^-^. [**1**] = 20 μM, [I^-^] = 0 to 100 μM. I^-^ exists as the (*n*-Bu)_4_N^+^ salt. The increased absorbance range from 200 to 270 nm comes from the absorption of I^-^.


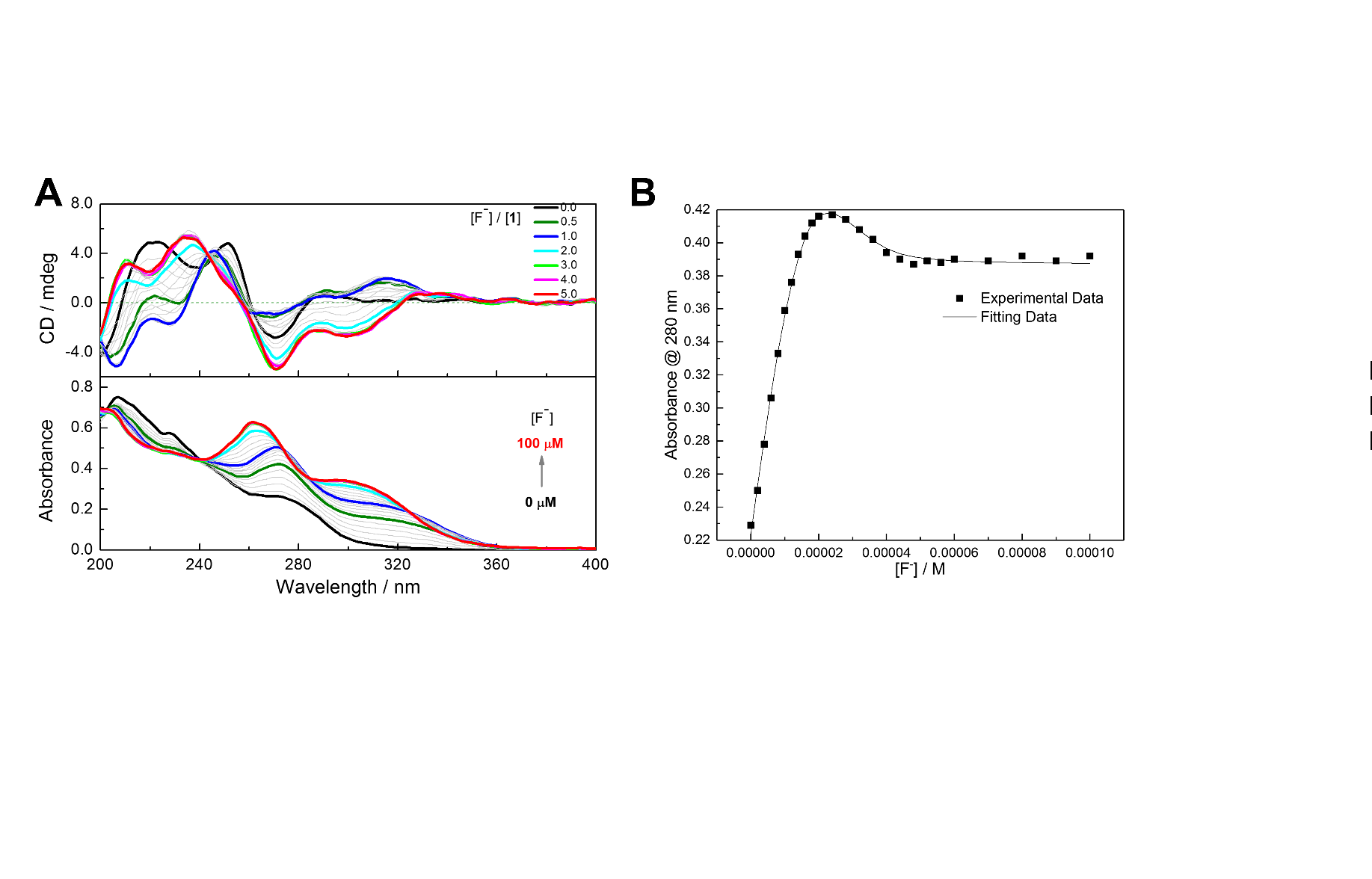


**Supplementary Figure S13.** (**A**) Absorption and CD spectra of **1** in CH_3_CN in the presence of F^-^. [**1**] = 20 μM, [F^-^] = 0 to 100 μM. F^-^ exists as the (*n*-Bu)_4_N^+^ salt. (**B**) Plots of absorbance (280 nm) against the concentration of F^-^ and the fitting curve, showing K_21_ = 1.7 × 10^5^ M^-1^, K_11_ = 1.9 × 10^8^ M^-1^, K_12_ = 8.6 × 10^5^ M^-1^. It is noted that K_11_ > K_12_, presumably due to the existence of the electronic repulsion in the 1:2 binding complex.


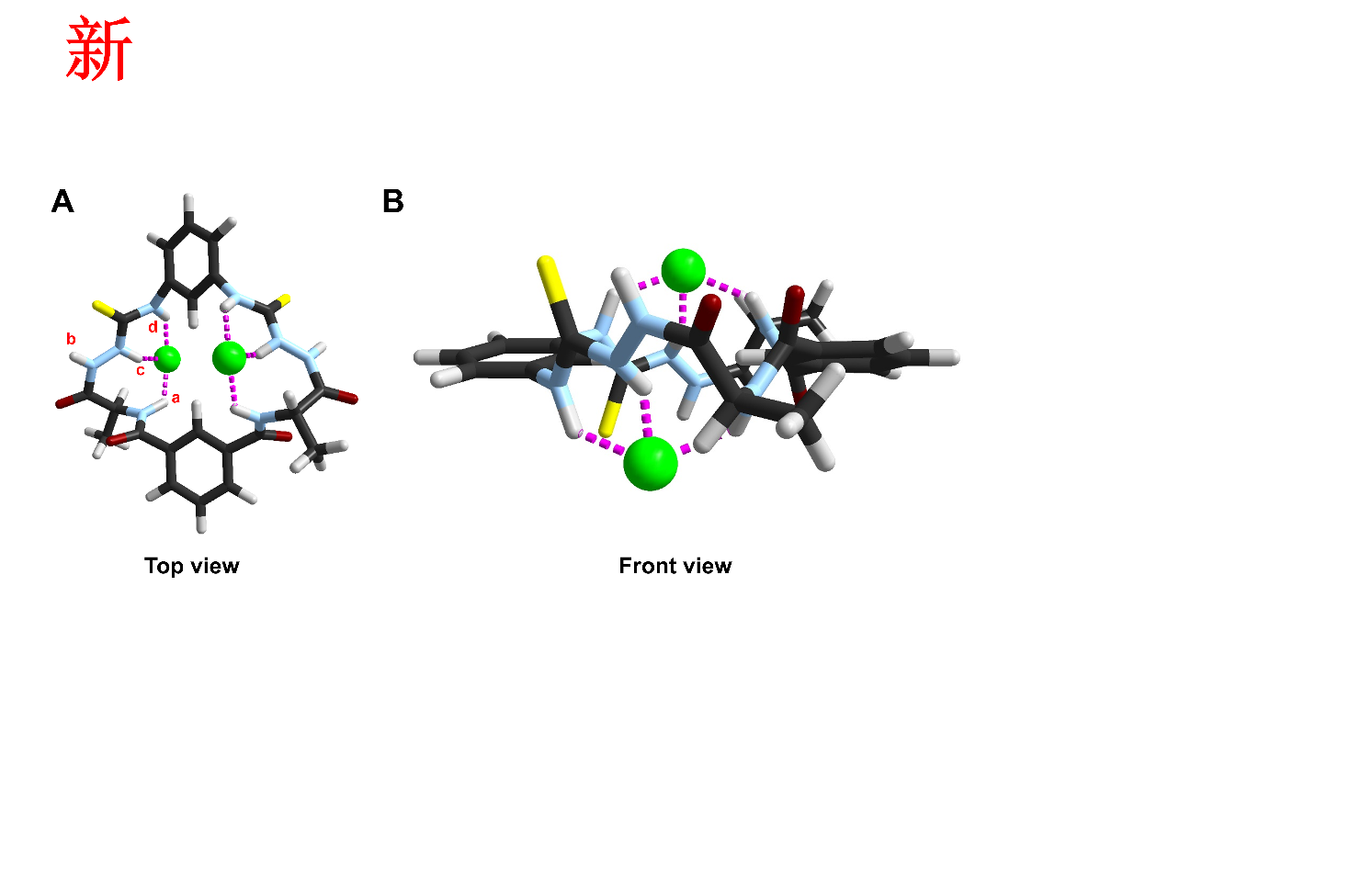


**Supplementary Figure S14.** Optimized structure of **1**∙2F^-^ (**A**, top view; **B**, front view) by B3LYP-D3(BJ)/6-311G. Dashed pink lines highlight intermolecular hydrogen bonds. The two F^-^ ions point to up and down, respectively, to reduce the electronic repulsion.

**Supplementary Table S2.** Parameters for N–H∙∙∙F^-^ hydrogen bonds in the optimized structure of **1**∙2F^-^ complex *^a^*

| Hydrogen bond | Length of H∙∙∙F^-^ / Å | Length of N∙∙∙F^-^ / Å | Angle of N–H∙∙∙F^-^ / º |
| --- | --- | --- | --- |
| N–H_a_∙∙∙F^-^ | 2.037 | 2.814 | 125.63 |
| N–H_c_∙∙∙F^-^ | 1.751 | 2.520 | 140.65 |
| N–H_d_∙∙∙F^-^ | 1.997 | 2.852 | 130.05 |

*^a^* The optimized structure of **1**∙2F^-^ complex is of C2 symmetry, so the hydrogen bonds at the two sides are the same.





**Supplementary Figure S15.** The experimental and calculated CD spectra of **1**∙2F^-^ in CH_3_CN solutions. TD-DFT with CAM-B3LYP/6-311G.


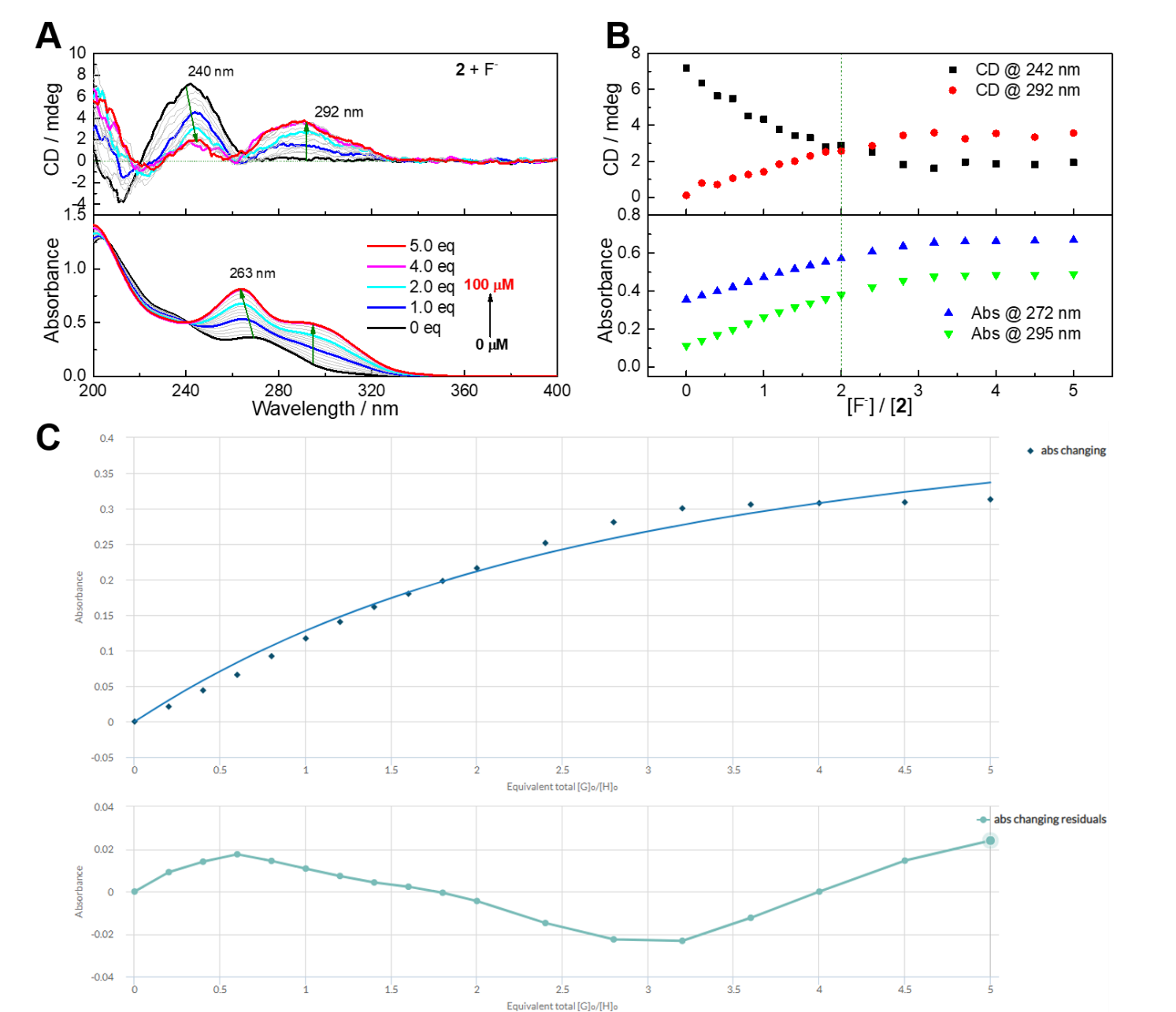


**Supplementary Figure S16.** (**A**) Absorption and CD spectra of **2** in CH_3_CN in the presence of F^-^. [**2**] = 20 μM, [F^-^] = 0 to 100 μM. F^-^ exists as the (*n*-Bu)_4_N^+^ salt. (**B**) Plots of CD intensity (242 nm, 292 nm) and absorbance (272 nm, 295 nm) against the concentration of F^-^. (**C**) Fitting results (absorbance at 272 nm, K = 2.2 × 10^5^ M^-1^) calculated using global fitting analysis from supramolecular.org (http://www.supramolecular.org, accessed Oct 24, 2020). One process is revealed from the titrations, so the fitting is based on the 1:1 model.


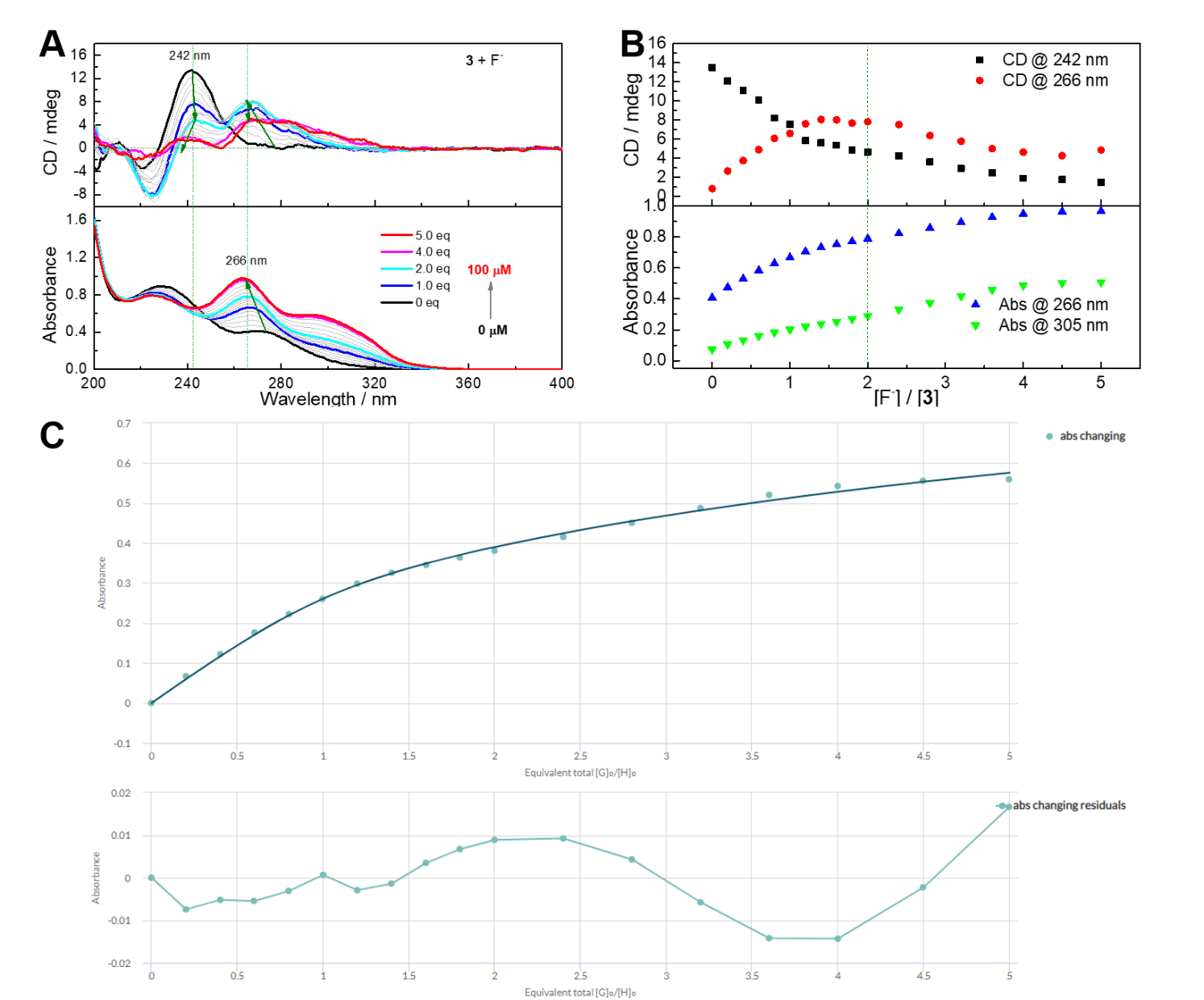


**Supplementary Figure S17.** (**A**) Absorption and CD spectra of **3** in CH_3_CN in the presence of F^-^. [**3**] = 20 μM, [F^-^] = 0 to 100 μM. F^-^ exists as the (*n*-Bu)_4_N^+^ salt. (**B**) Plots of CD intensity (242 nm, 266 nm) and absorbance (266 nm, 305 nm) against the concentration of F^-^. (**C**) Fitting results (absorbance at 266 nm, K_11_ = 5.9 × 10^5^ M^-1^, K_12_ = 8.5 × 10^3^ M^-1^) calculated using global fitting analysis from supramolecular.org (http://www.supramolecular.org, accessed Oct 24, 2020). Two processes are revealed from the titrations, so the fitting is based on the 1:2 model.


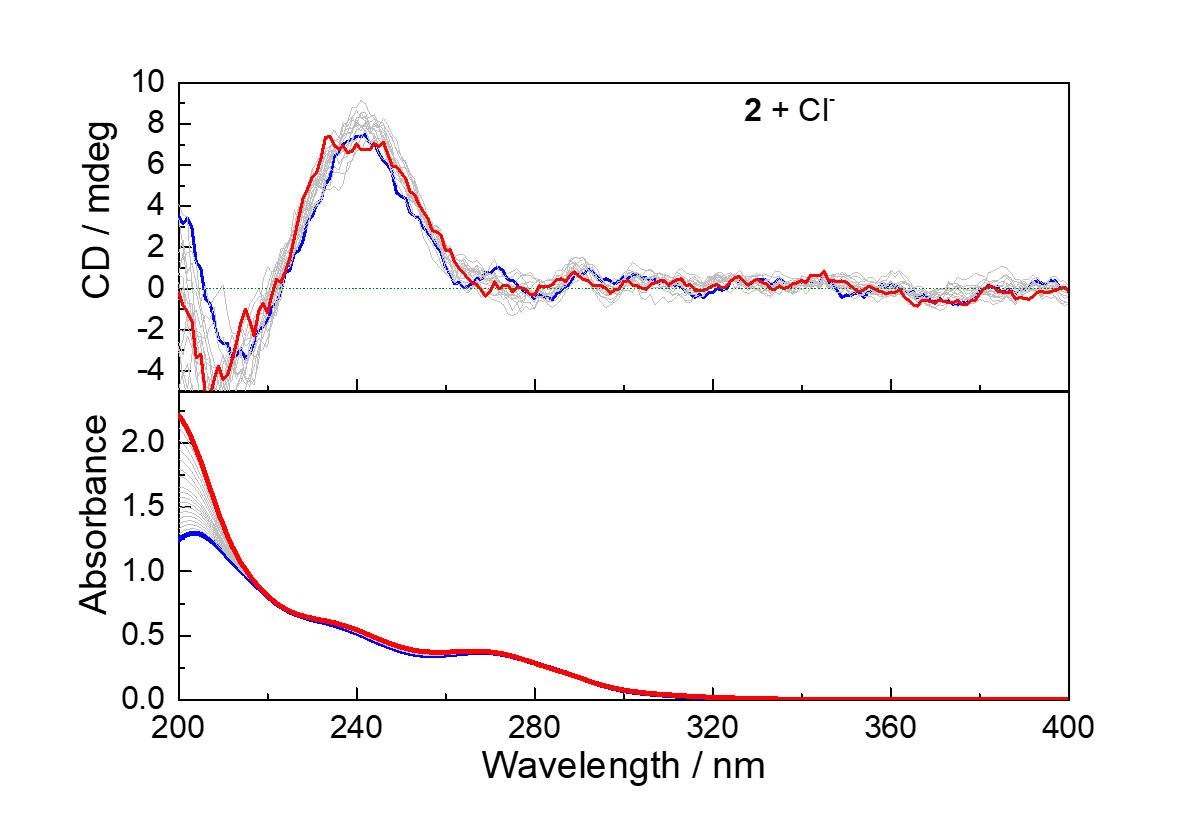


**Supplementary Figure S18.** Absorption and CD spectra of **2** in CH_3_CN in the presence of Cl^-^. [**2**] = 20 μM, [Cl^-^] = 0 to 100 μM. Cl^-^ exists as the (*n*-Bu)_4_N^+^ salt. The increased absorbance range from 200 to 220 nm comes from the absorption of Cl^-^.


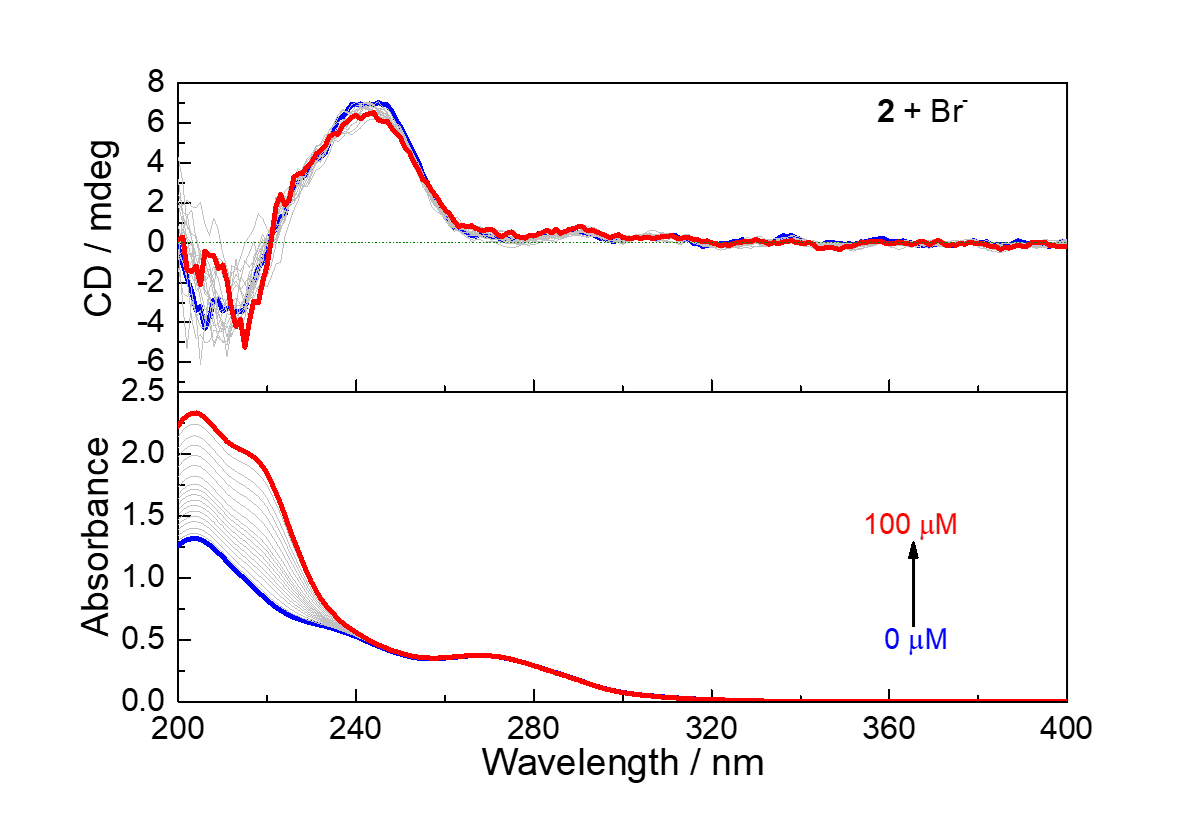


**Supplementary Figure S19.** Absorption and CD spectra of **2** in CH_3_CN in the presence of Br^-^. [**2**] = 20 μM, [Br^-^] = 0 to 100 μM. Br^-^ exists as the (*n*-Bu)_4_N^+^ salt. The increased absorbance range from 200 to 240 nm comes from the absorption of Br^-^.


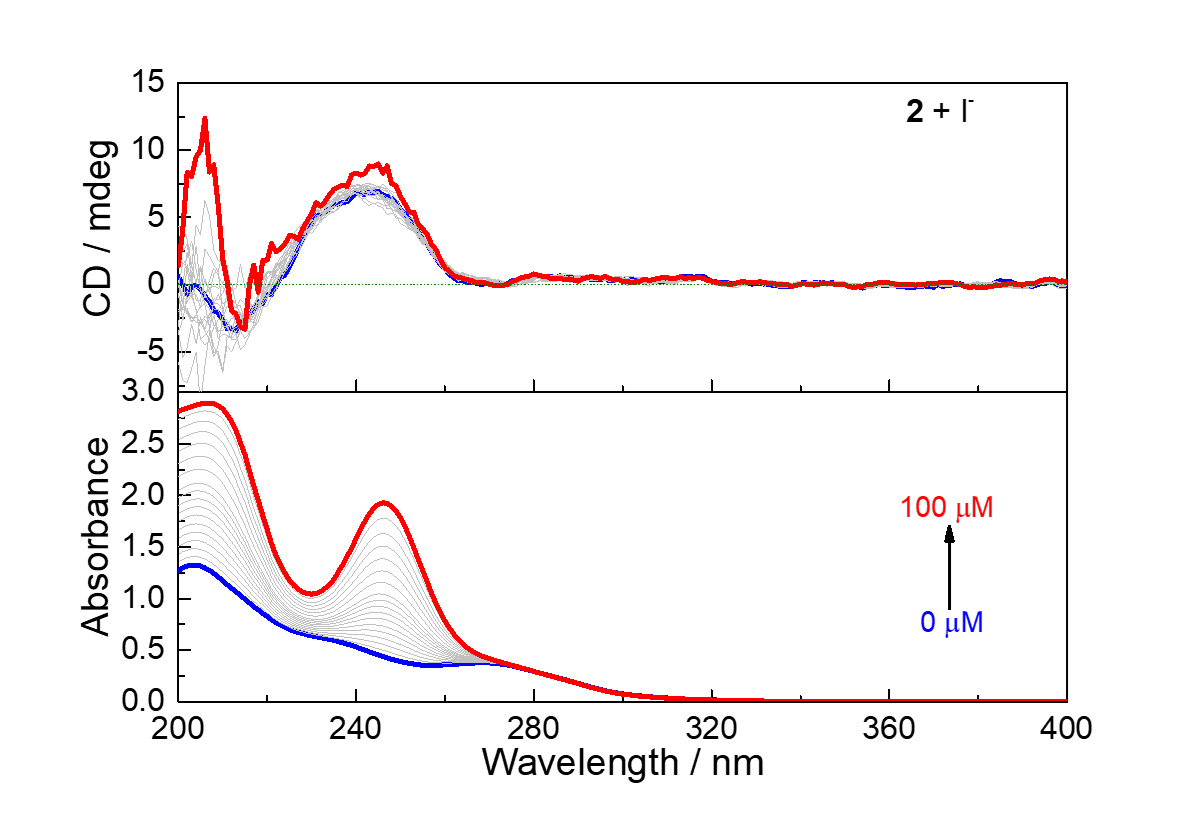


**Supplementary Figure S20.** Absorption and CD spectra of **2** in CH_3_CN in the presence of I^-^. [**2**] = 20 μM, [I^-^] = 0 to 100 μM. I^-^ exists as the (*n*-Bu)_4_N^+^ salt. The increased absorbance range from 200 to 270 nm comes from the absorption of I^-^.


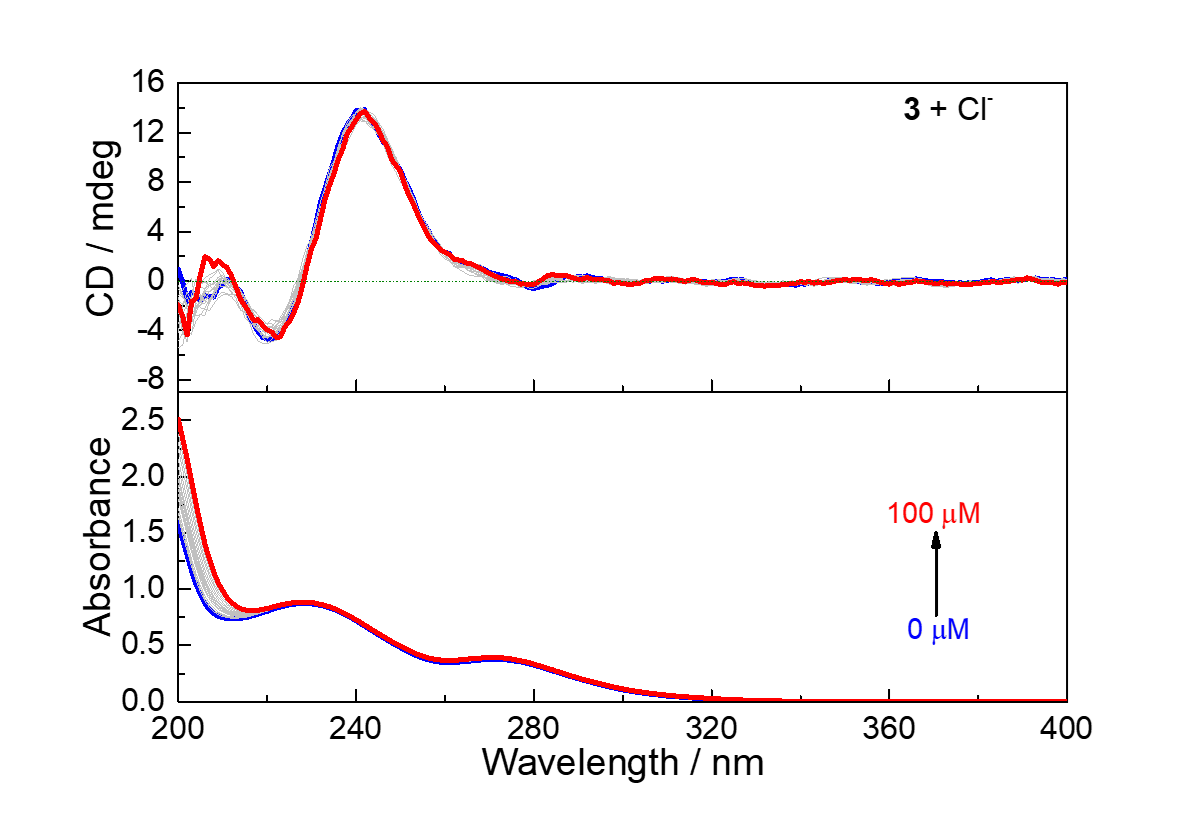


**Supplementary Figure S21.** Absorption (A) and CD (B) spectra of **3** in CH_3_CN in the presence of Cl^-^. [**3**] = 20 μM, [Cl^-^] = 0 to 100 μM. Cl^-^ exists as the (*n*-Bu)_4_N^+^ salt. The increased absorbance range from 200 to 220 nm comes from the absorption of Cl^-^.


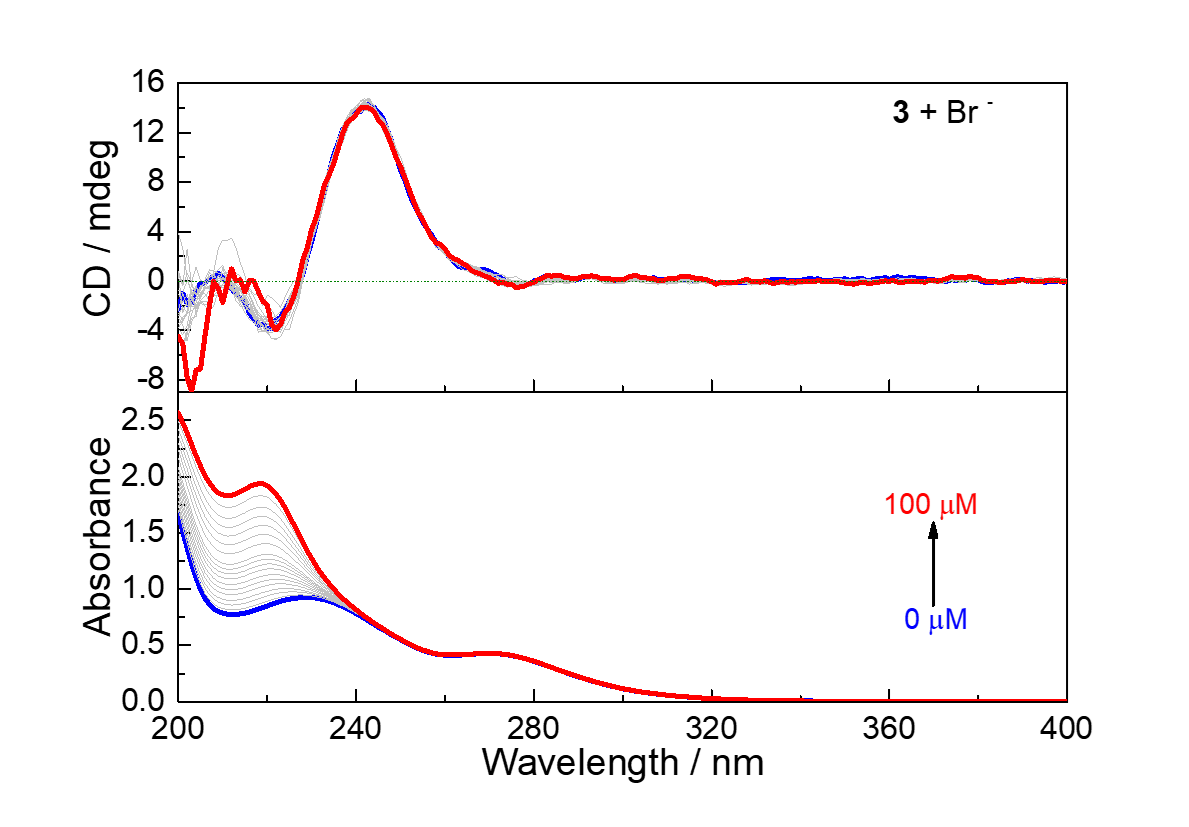


**Supplementary Figure S22.** Absorption (A) and CD (B) spectra of **3** in CH_3_CN in the presence of Br^-^. [**3**] = 20 μM, [Br^-^] = 0 to 100 μM. Br^-^ exists as the (*n*-Bu)_4_N^+^ salt. The increased absorbance range from 200 to 240 nm comes from the absorption of Br^-^.


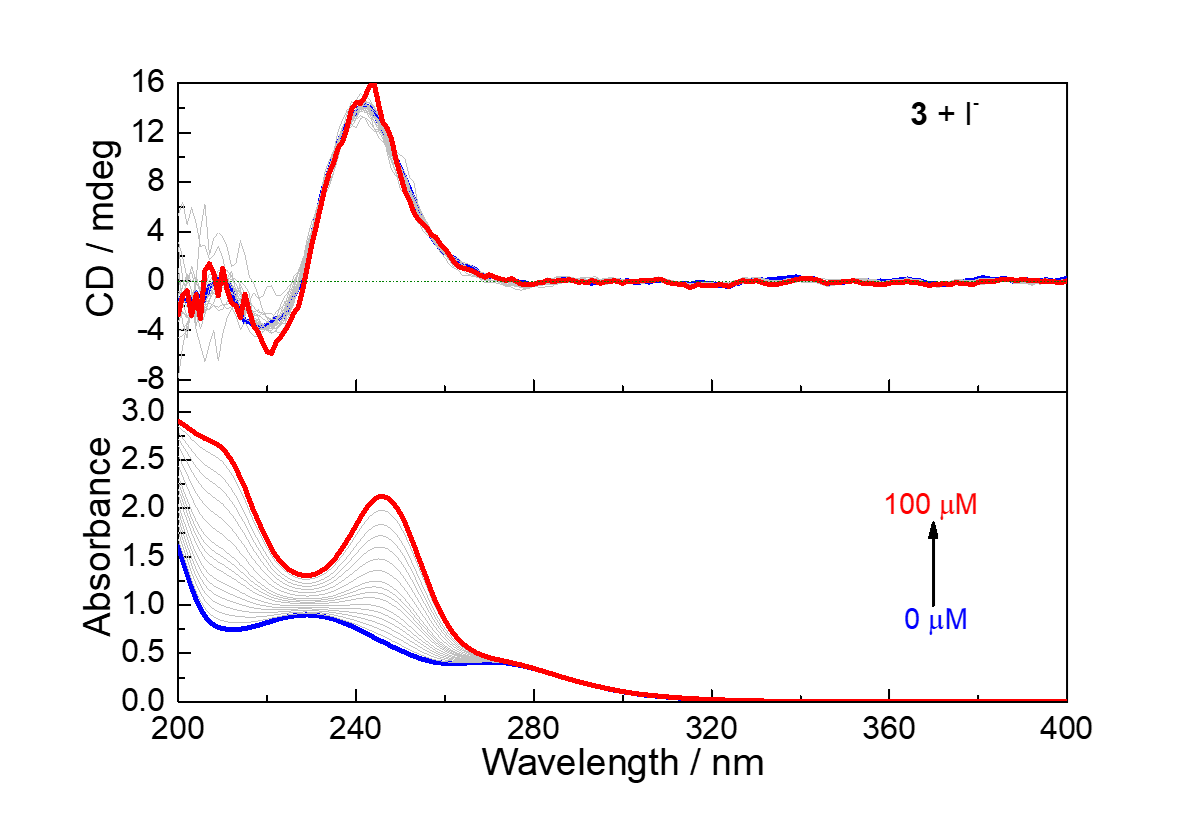


**Supplementary Figure S23.** Absorption (A) and CD (B) spectra of **3** in CH_3_CN in the presence of I^-^. [**3**] = 20 μM, [I^-^] = 0 to 100 μM. I^-^ exists as the (*n*-Bu)_4_N^+^ salt. The increased absorbance range from 200 to 270 nm comes from the absorption of I^-^.


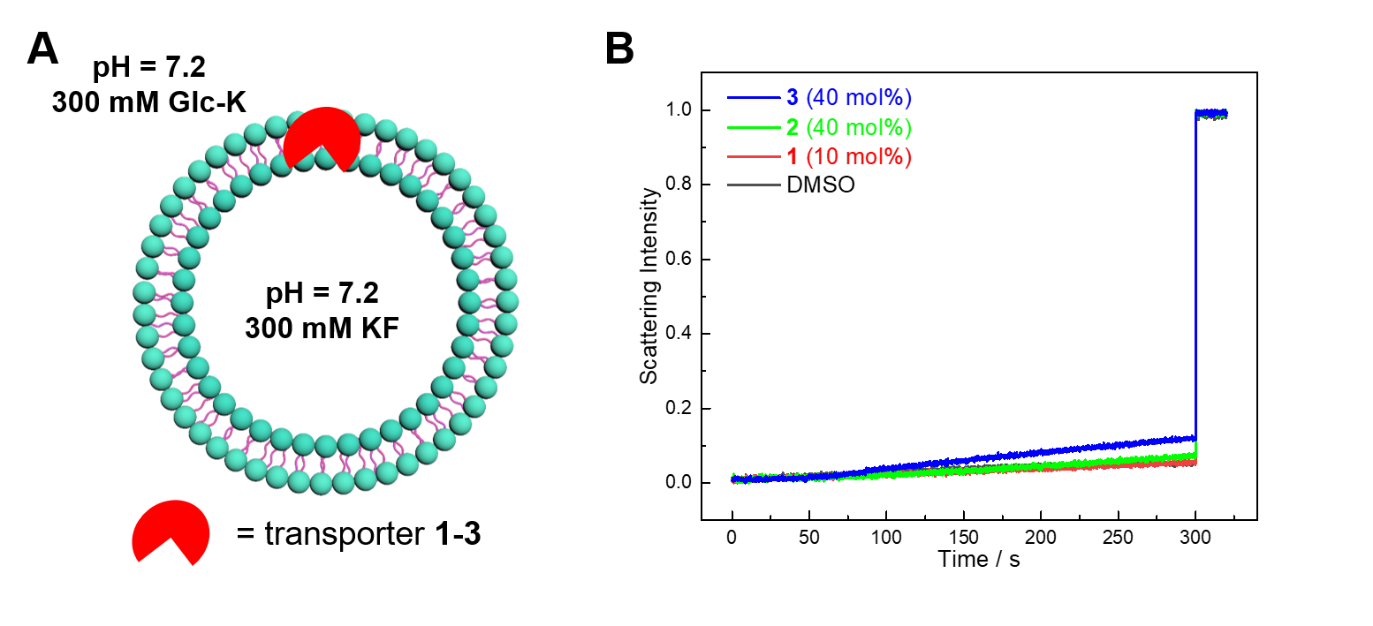


**Supplementary Figure S24. (A)** Schematic representation of osmotic assay for fluoride transport by exerting a concentration gradient of fluoride. Inside LUVs: 300 mM KF, 10 mM HEPES, pH 7.2. Outside LUVs: 300 mM Glc-K, 10 mM HEPES, pH 7.2. **(B)** Normalized fluorescence intensity obtained by addition of compounds **1** (50 μM, 10 mol%) and **2-3** (200 μM, 40 mol%).


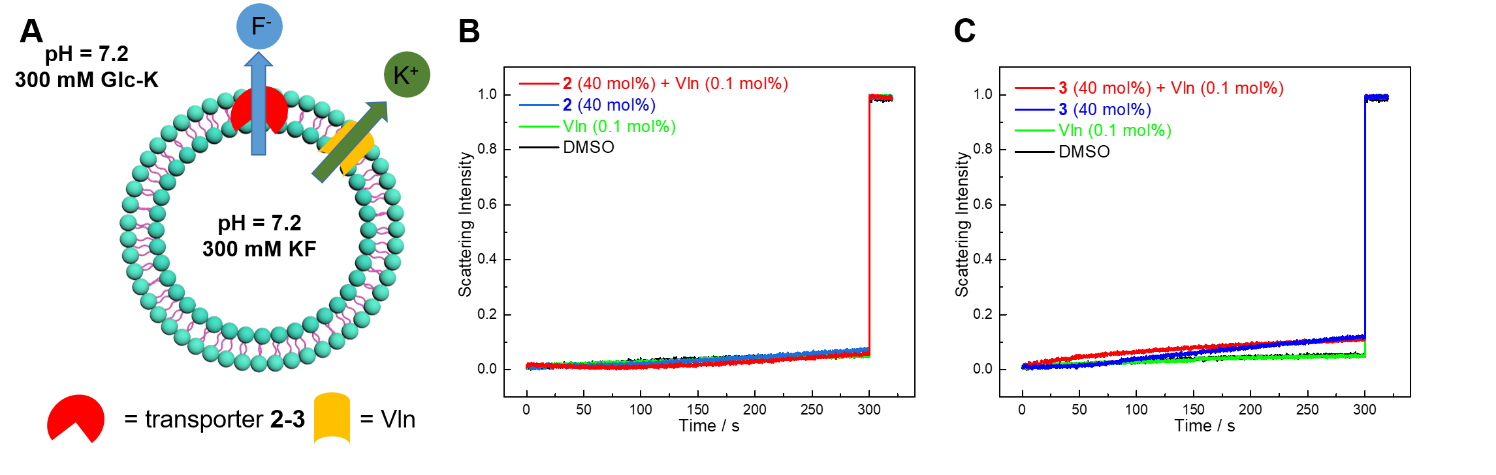


**Supplementary Figure S25. (A)** Schematic representation of osmotic assay for fluoride transport by exerting a concentration gradient of fluoride. Inside LUVs: 300 mM KF, 10 mM HEPES, pH 7.2. Outside LUVs: 300 mM Glc-K, 10 mM HEPES, pH 7.2. **(B,C)** Normalized fluorescence intensity obtained by addition of compounds **2** (200 μM, 40 mol%, B) and **3** (200 μM, 40 mol%, C) in the presence and absence of Vln (0.5 μM, 0.1 mol%).


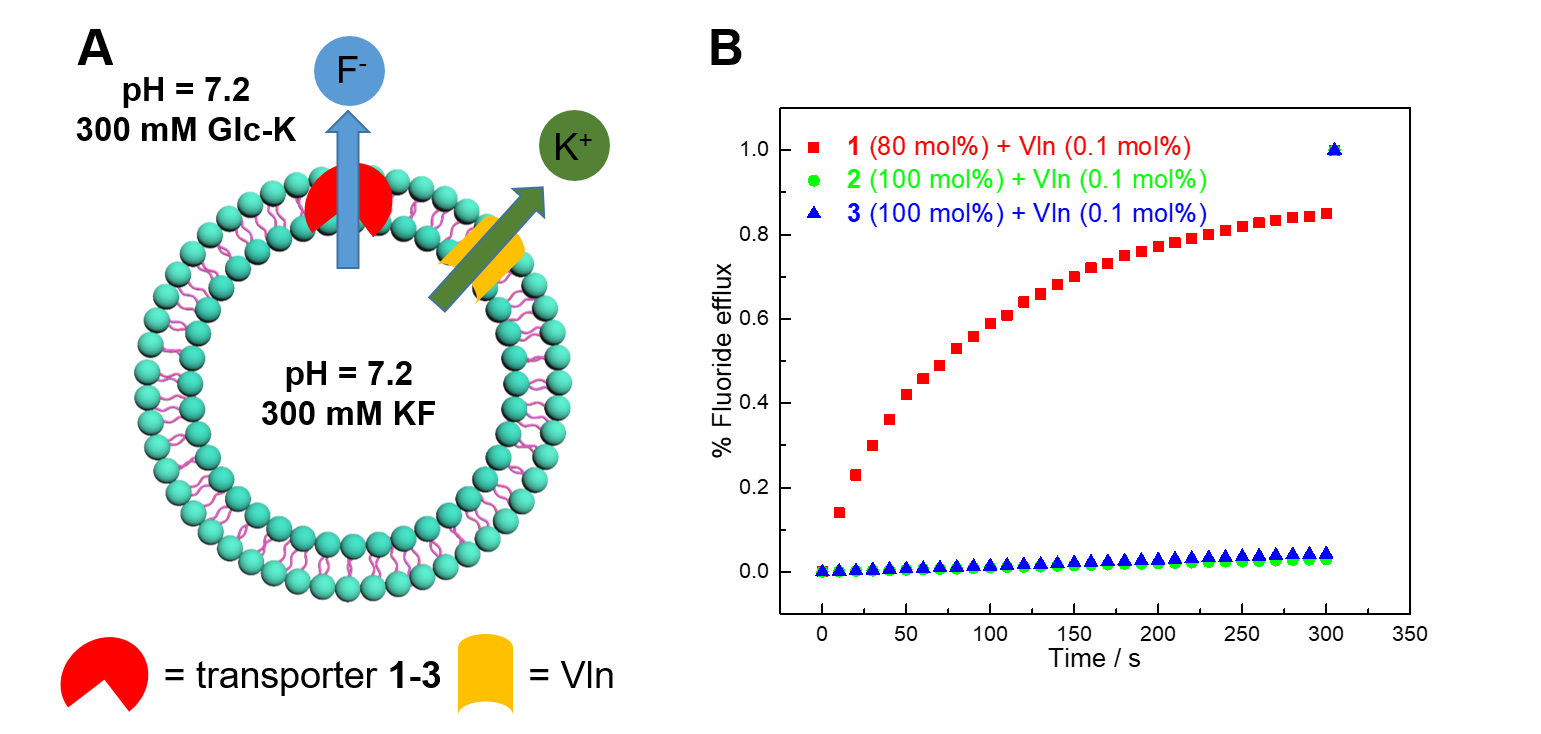


**Supplementary Figure S26. (A)** Schematic representation of ISE assay for fluoride transport by exerting a concentration gradient of fluoride. Inside LUVs: 300 mM KF, 10 mM HEPES, pH 7.2. Outside LUVs: 300 mM Glc-K, 10 mM HEPES, pH 7.2. **(B)** Normalized fluoride efflux obtained by addition of compounds **1** (80 mol%) and **2-3** (100 mol%) coupled with Vln (0.5 μM, 0.1 mol%).


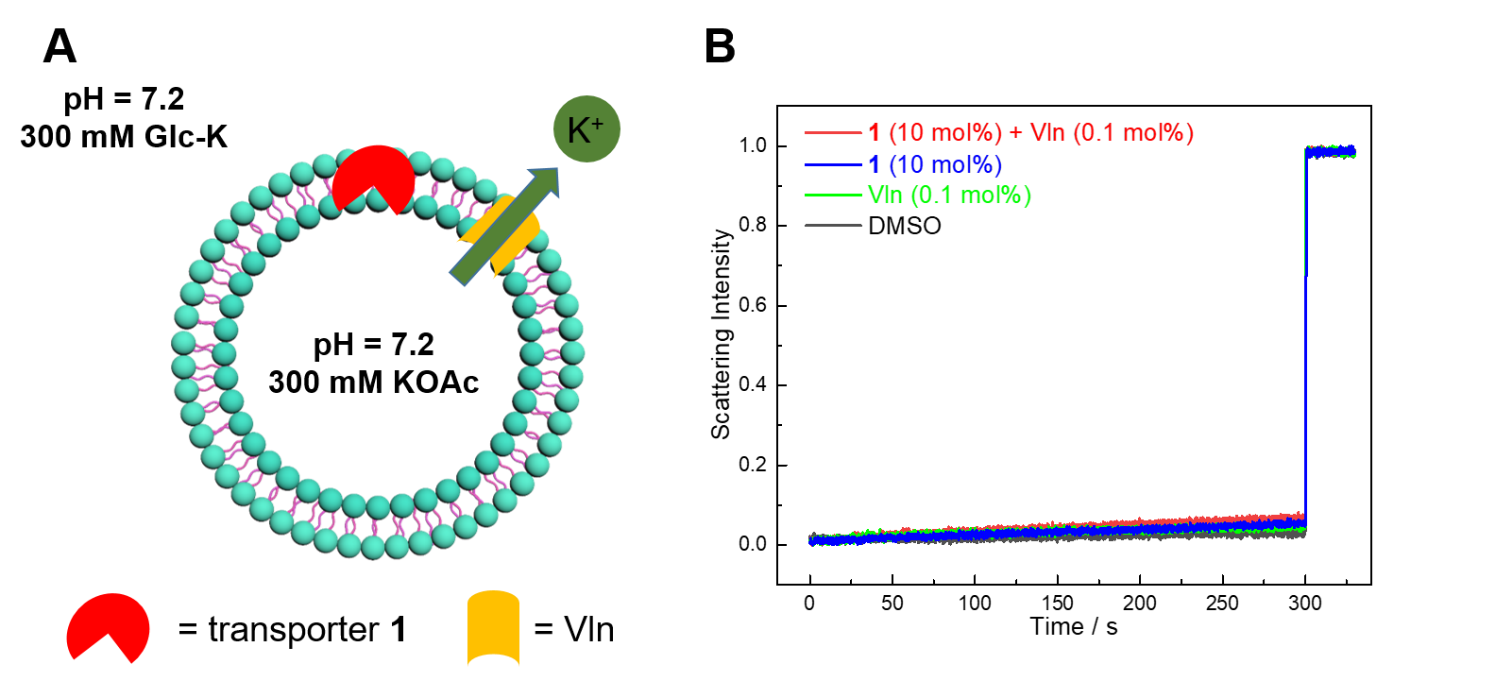


**Supplementary Figure S27. (A)** Schematic representation of osmotic assay for acetate ion transport by exerting a concentration. Inside LUVs: 300 mM KOAc, 10 mM HEPES, pH 7.2. Outside LUVs: 300 mM Glc-K, 10 mM HEPES, pH 7.2. **(B)** Normalized fluorescence intensity obtained by addition of compounds **1** (50 μM, 10 mol%) in the presence and absence of Vln (0.5 μM, 0.1 mol%).


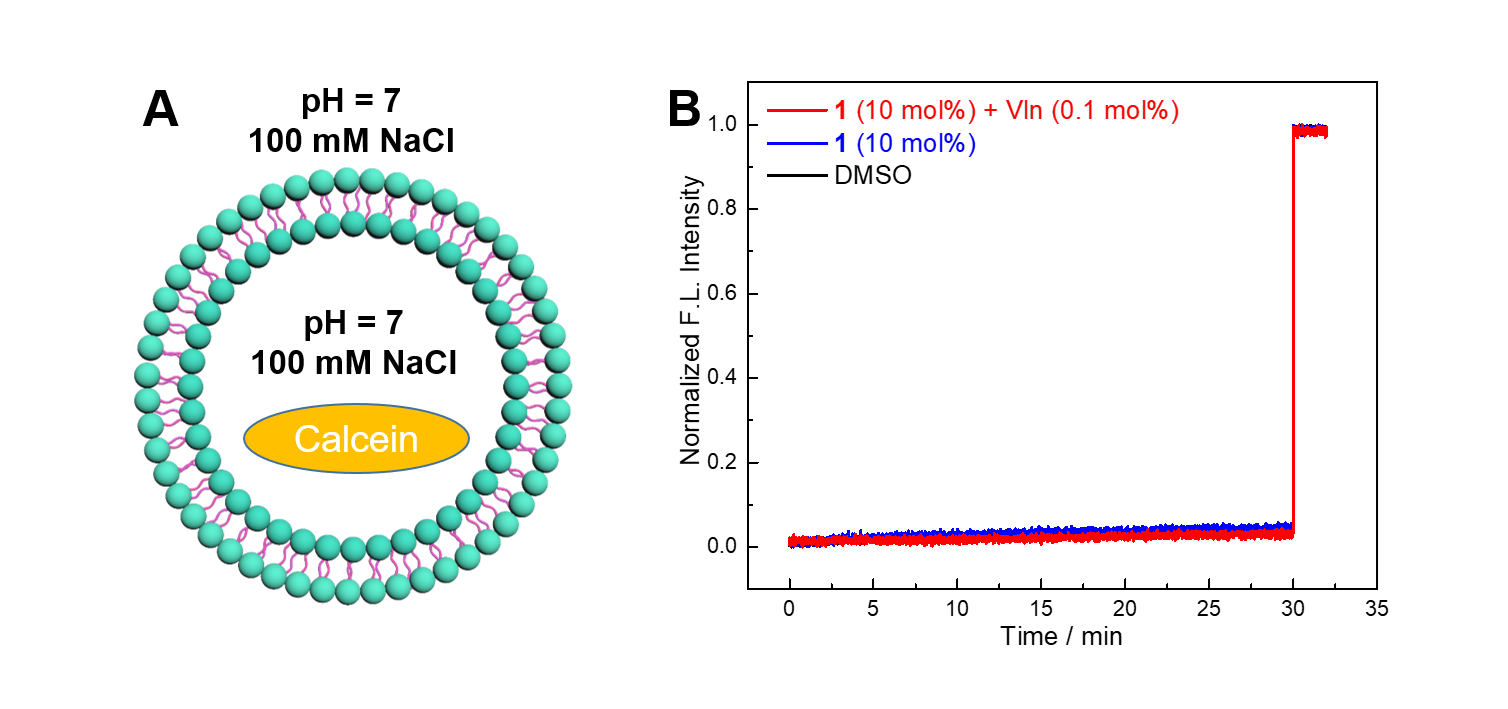


**Supplementary Figure S28.** **(A)** Schematic representation of calcein leakage assay with time in the presence of various transporters. **(B)** Normalized fluorescence intensity obtained by addition of **1** (10 mol%) coupled with Vln (0.1 mol%).


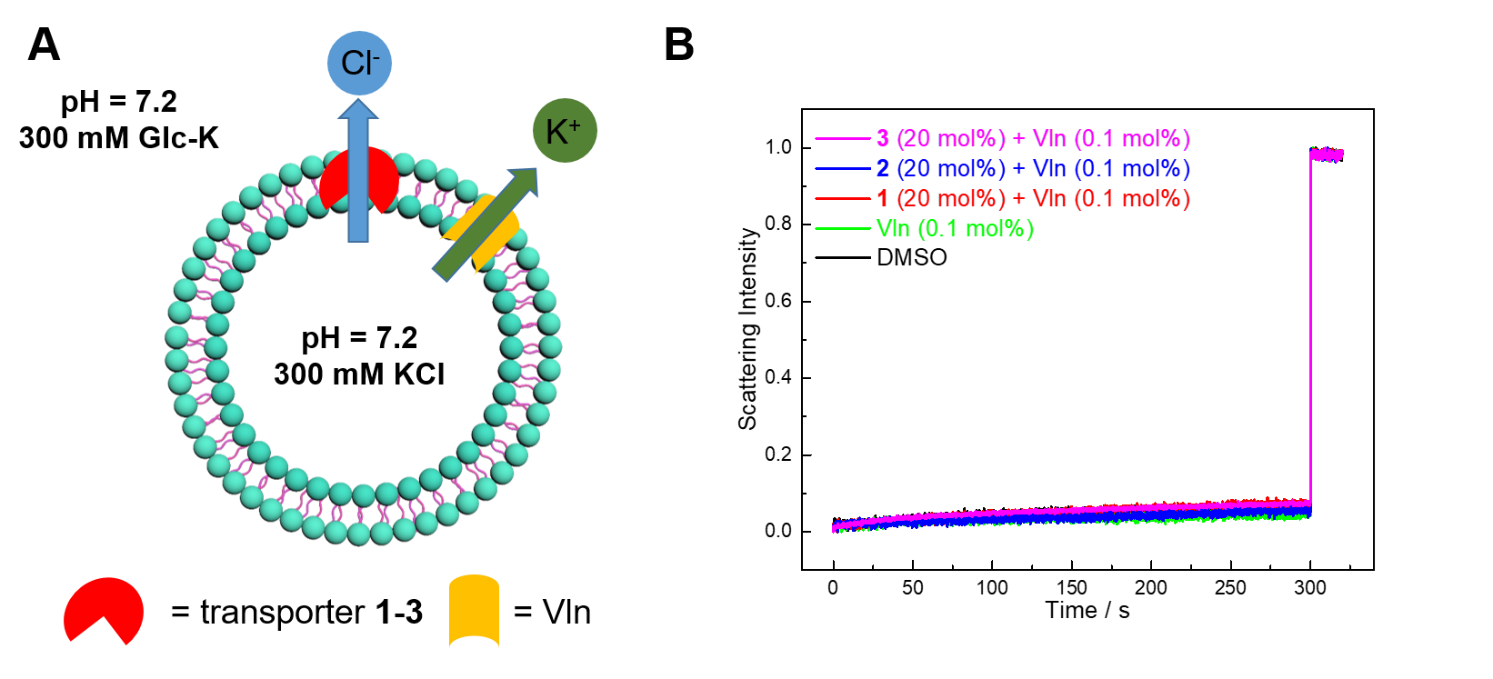


**Supplementary Figure S29. (A)** Schematic representation of osmotic assay for chloride transport by exerting a concentration gradient of chloride. Inside LUVs: 300 mM KCl, 10 mM HEPES, pH 7.2. Outside LUVs: 300 mM Glc-K, 10 mM HEPES, pH 7.2. **(B)** Normalized fluorescence intensity obtained by addition of compounds **1-3** (20 mol%) coupled with Vln (0.5 μM, 0.1 mol%)..


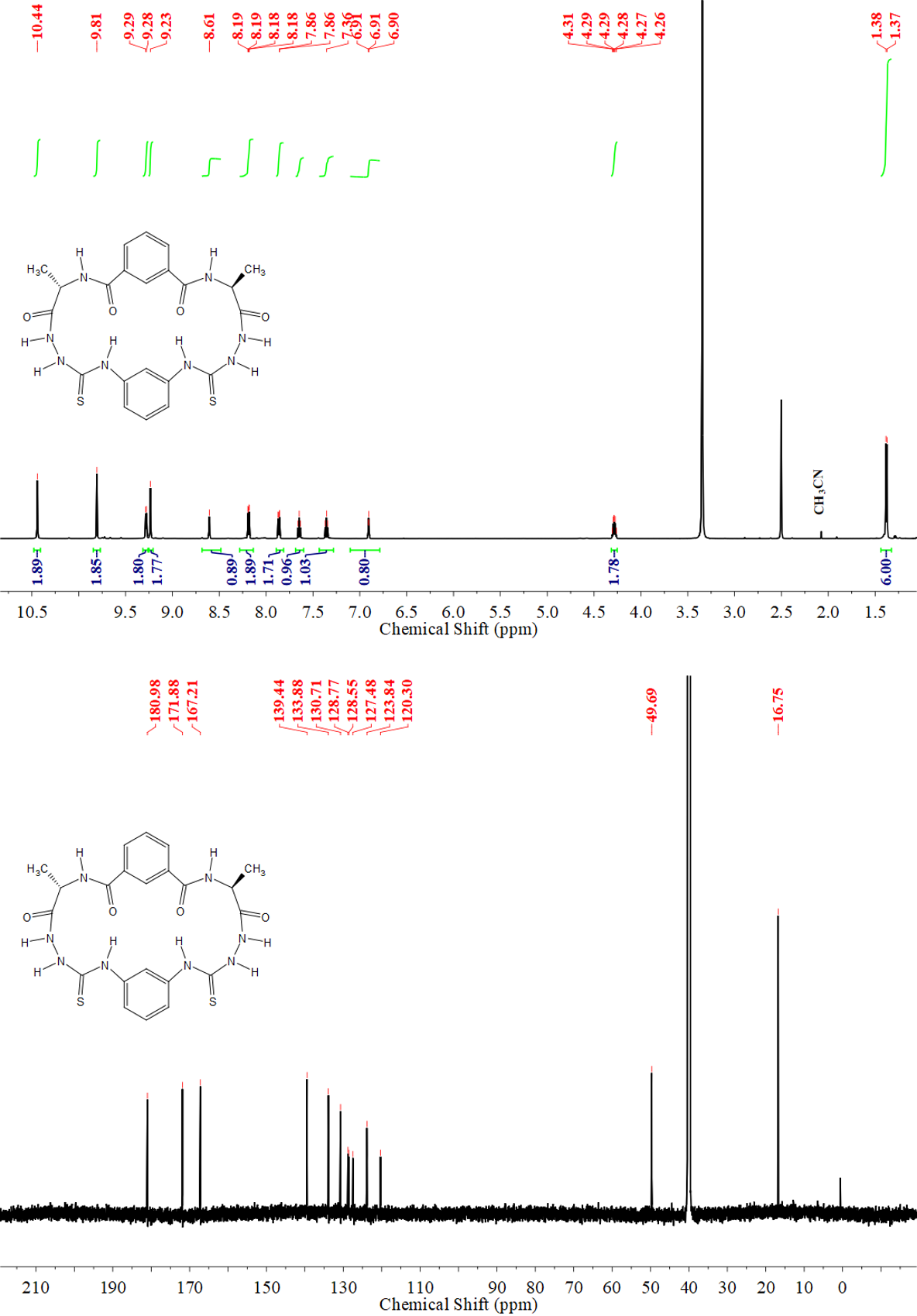


**Supplementary Figure S30.** ^1^H and ^13^C NMR spectra of **1**.


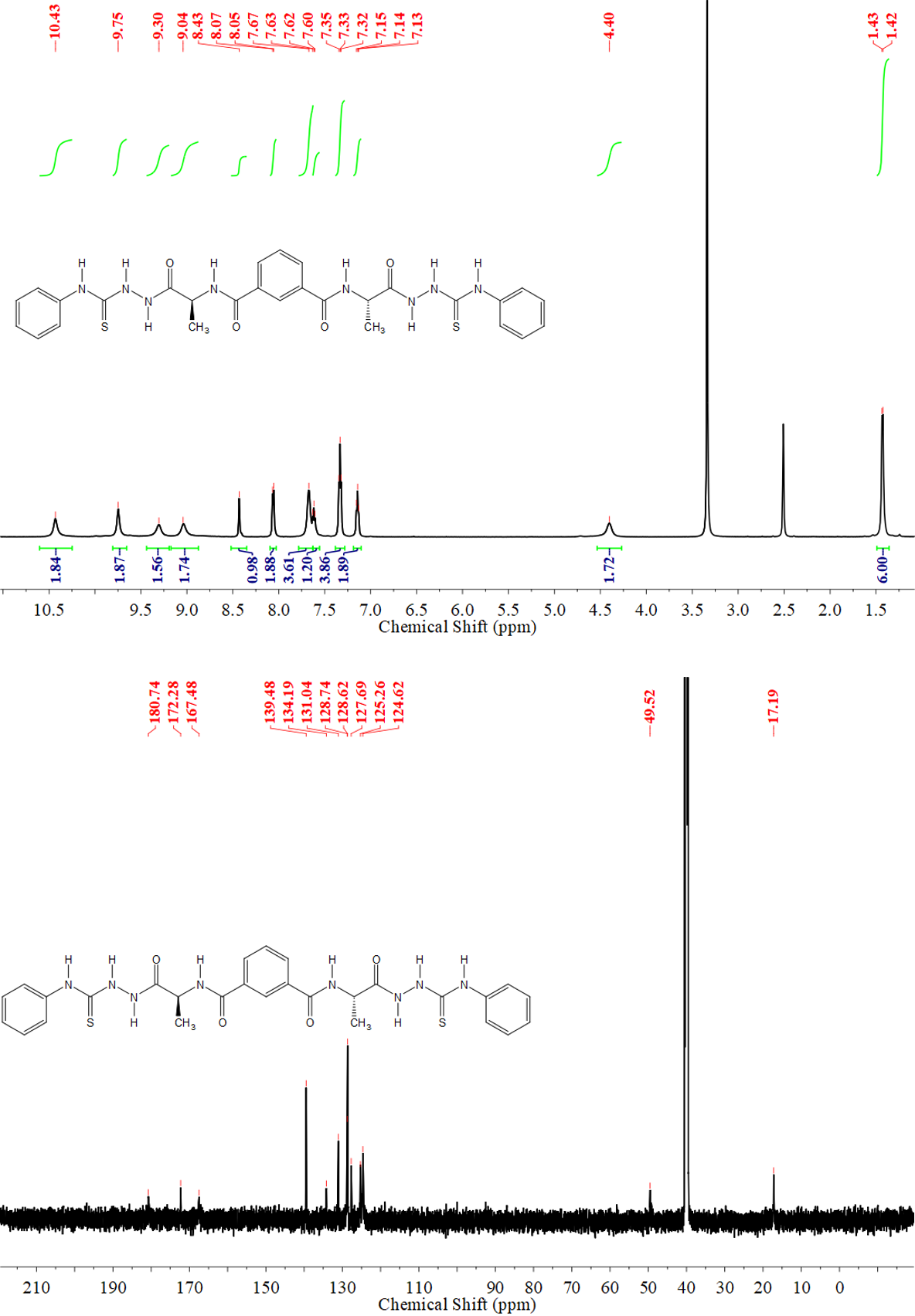


**Supplementary Figure S31**. ^1^H and ^13^C NMR spectra of **2**.


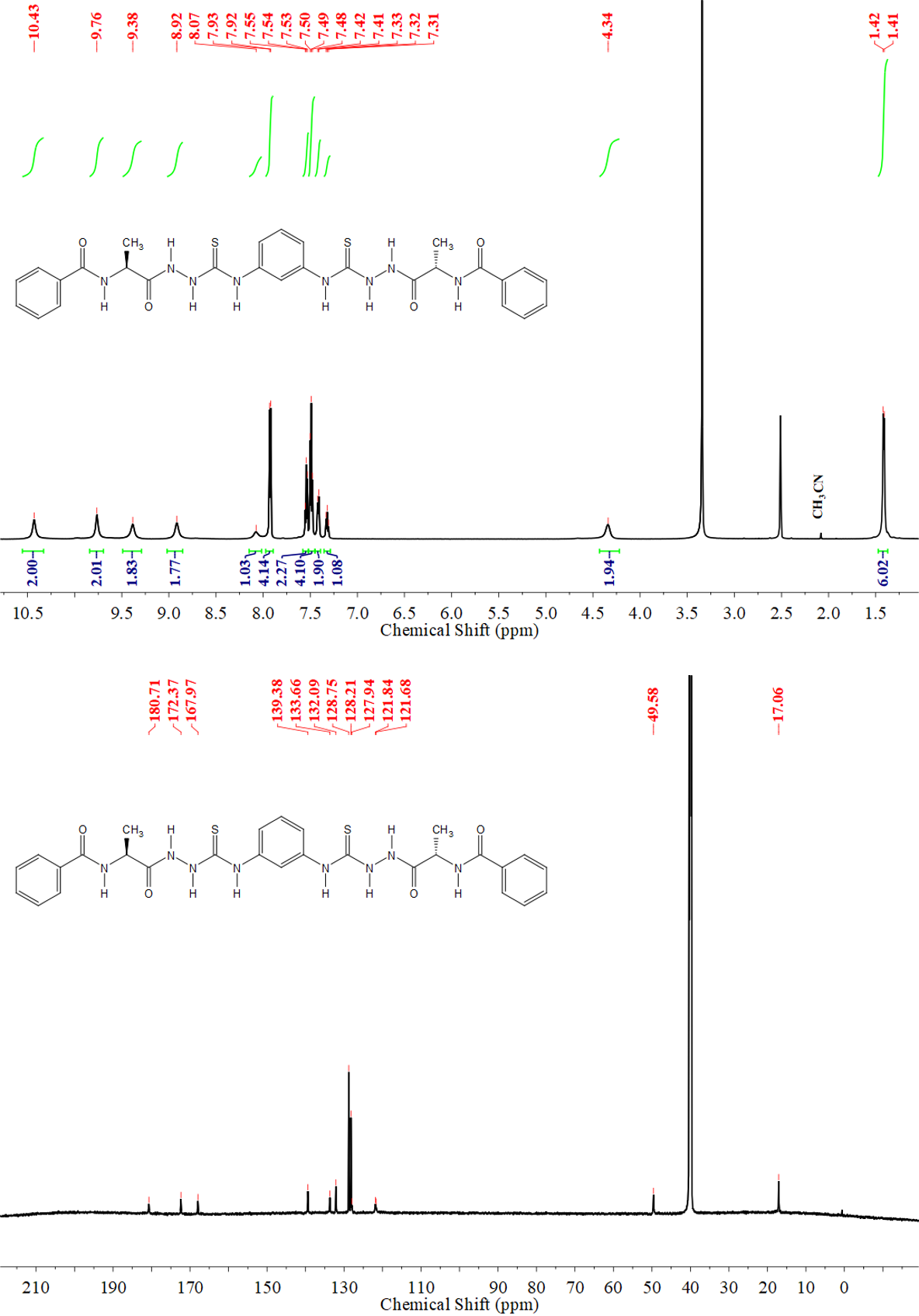


**Supplementary Figure S32**. ^1^H and ^13^C NMR spectra of **3**.


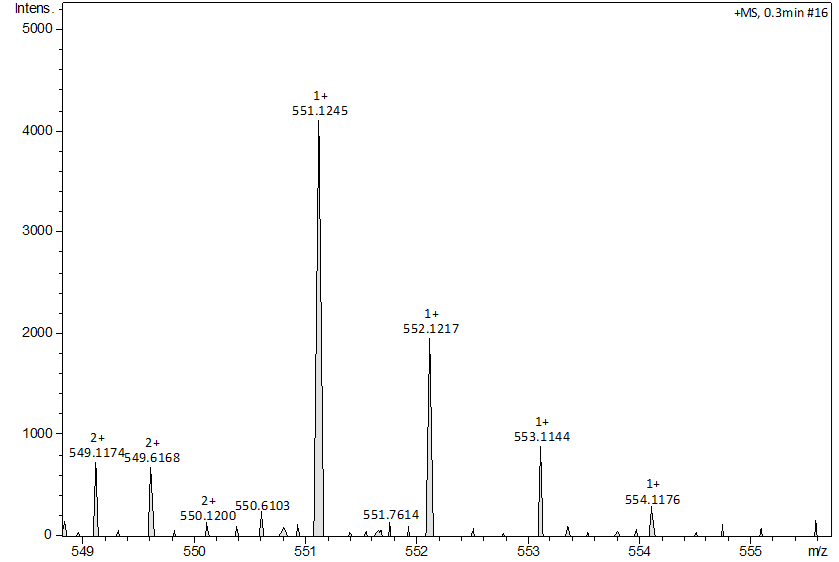


**Supplementary Figure S33.** HRMS (ESI-MS) of **1**.


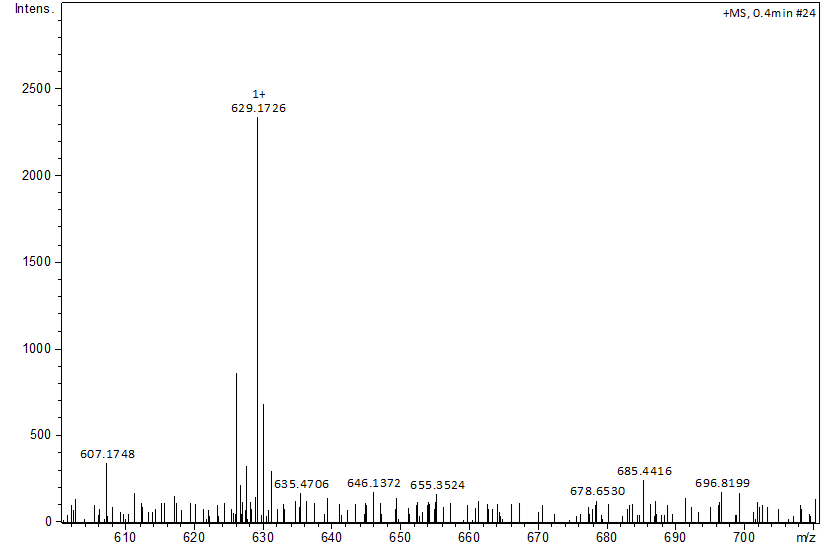


**Supplementary Figure S34.** HRMS (ESI-MS) of **2**.


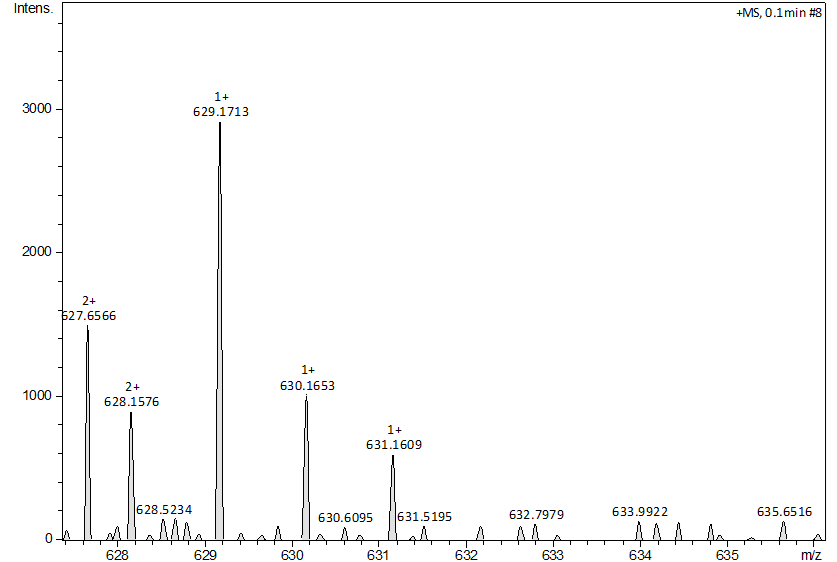


**Supplementary Figure S35.** HRMS (ESI-MS) of **3**.
